# A multi-event capture-recapture analysis of *Toxoplasma gondii* seroconversion dynamics in farm cats

**DOI:** 10.1186/s13071-018-2834-4

**Published:** 2018-06-08

**Authors:** Julie Alice Simon, Roger Pradel, Dominique Aubert, Régine Geers, Isabelle Villena, Marie-Lazarine Poulle

**Affiliations:** 1 Université de Reims Champagne-Ardenne, Laboratoire de Parasitologie – Mycologie, EA 3800, UFR Médecine, SFR CAP-SANTÉ, 51 rue Cognacq Jay, 51095 Reims, France; 2 Université de Reims Champagne-Ardenne, Centre d’Etude et de Formation en Eco-Ethologie (URCA, CERFE), 5 rue de la Héronnière, 08240 Boult-aux-Bois, France; 3 Centre d’Ecologie Fonctionnelle et Evolutive (CEFE) UMR 5175, CNRS – Université de Montpellier - Université Paul Valéry – EPHE, 1919 route de Mende, 34293 Montpellier 5, France; 4 Laboratoire de Parasitologie-Mycologie, Centre National de Référence de la Toxoplasmose, Hôpital Maison Blanche, CHU Reims, 45 rue Cognacq Jay, 51092 Reims, France

**Keywords:** Toxoplasmosis, Domestic cats, Capture-recapture, Multi-event modelling, Infection dynamics, Serological titres, Blotting paper, Misclassification

## Abstract

**Background:**

Domestic cats play a key role in the epidemiology of the parasite *Toxoplasma gondii* by excreting environmentally-resistant oocysts that may infect humans and other warm-blooded animals. The dynamics of *Toxoplasma gondii* seroconversion, used as a proxy for primo-infection dynamics, was investigated in five cat populations living on farms.

**Methods:**

Serological tests on blood samples from cats were performed every three months over a period of two years, for a total of 400 serological tests performed on 130 cats. Variations in seroconversion rates and associated factors were investigated using a multi-event capture-recapture modelling approach that explicitly accounted for uncertainties in cat age and serological status.

**Results:**

Seroprevalence varied between farms, from 15 to 73%, suggesting differential exposure of cats to *T. gondii*. In farms with high exposure, cats could become infected before reaching the age of six months. Seroconversion rates varied from 0.42 to 0.96 seroconversions per cat per year and were higher in autumn and winter than in spring and summer.

**Conclusion:**

Our results suggest inter-farm and seasonal variations in the risks of exposure to *T. gondii* oocysts for humans and livestock living on farms. The paper also discusses the role of young cats in the maintenance of environmental contamination by *T. gondii* oocysts on farms.

**Electronic supplementary material:**

The online version of this article (10.1186/s13071-018-2834-4) contains supplementary material, which is available to authorized users.

## Background

Estimating parasitic infection rates in natural host populations often requires longitudinal surveys with repeated sampling of individuals in order to gather information on their infection history [1]. These surveys present methodological challenges and can result in a range of uncertainties that researchers must deal with. The first source of uncertainty is inherent to longitudinal surveys, as a previously sampled individual may not be detected, captured or tested at each sampling occasion, resulting in partial observations of its infectious states over time (i.e. incomplete infection history; [2]). A second source of uncertainty may result from the lack of reliability of certain diagnostic tests or observations in assigning the infectious state of an individual: a given state may be assigned erroneously (false positive or negative), resulting in misclassification and mistaken interpretations [2–4]. These are particularly encountered when pathogen exposure is inferred from qualitative measures, such as the presence of specific antibodies [5]. The classification of an individual as ‘infected’ or ‘susceptible’ then results in an approximation of the continuous distribution of the immune response [6, 7].

Recently, modelling methodologies used for population dynamics, such as site-occupancy modelling [4, 8] and multi-event capture-mark-recapture (CMR) [9], have been proposed to deal with this state uncertainty issue in epidemiologic studies. Multi-event CMR approach especially allows the estimation of infection rates corrected by individuals detection probabilities by relating the way an individual is observed and recorded in the field (observational process) to the unobserved biological states of individuals that is inferred by the model (biological process; [9–11]). Parameters of these two processes are estimated using probabilistic statements describing the probability of each possible detection and serological histories combined, based on the likelihood estimation of the whole histories of all the surveyed individuals. Multi-event capture-mark-recapture has especially been used to study the exposure dynamics of black-legged kittiwakes (*Rissa tridactyla*) to Lyme disease [6], the immune dynamic of European rabbits (*Oryctolagus cuniculus*) to myxoma and rabbit hemorrhagic disease virus [12] and more recently the probability of true *Mycobacterium bovis* infection in badger (*Meles meles*) [13]. In this study, we used this approach to investigate the dynamics of *Toxoplasma gondii* infection in farm cat populations.

*Toxoplasma gondii* is a ubiquitous parasite that causes toxoplasmosis in humans and other warm-blooded animals [14]. This protozoan is considered of high medical importance as it can cause severe illness that can be life-threatening in immunocompromised individuals or in foetuses when acquired congenitally [15]. *Toxoplasma gondii* also represents a veterinary issue as a major source of reproductive failure in small ruminants [16–18], and a cause of fatal infections in some wildlife species [19–21]. Wild and domestic felids are the only known definitive hosts of *T. gondii* and they play a major role in spreading the parasite by shedding oocysts in faeces. These oocysts become infectious after sporulation in the environment and can survive and remain infectious for months in soil and water [22–24]. Accidental ingestion of oocysts contained in water, soil and vegetables is a major source of infection for animal intermediate hosts [25] as well as for humans [26, 27].

Among definitive hosts, the domestic cat (*Felis silvestris catus*) plays a key role in the epidemiology of the parasite [14, 28], especially the free ranging cats settled in livestock farms (and so called “farm cats”), since these farms are considered as hot-spots of environmental contamination by *T. gondii* in rural areas [29–31]. It is believed that cats become infected most often through the consumption of a contaminated prey [32]. After a primary-infection, cats can excrete millions of *T. gondii* oocysts in their faeces over a period of 7–20 days and then develop a long-lasting humoral immune response against the parasite. Bradyzoites developed following infection may persist within cysts in tissues for the life of the host and IgG antibodies probably do as well [33]. In cats with reactivation of chronic toxoplasmosis from immune suppression, IgG titres only rarely increase [34].

Estimating primary infection rates in cats is directly relevant to predicting the oocyst burden in the environment, and thus in evaluating the infection risk to humans and animals. Most of the studies that have attempted to assess the determinants of infection by *T. gondii* in natural hosts populations were based on serological data by estimating seroprevalence, i.e. proportion of individuals within a population that demonstrate *T. gondii*-specific antibody in the serum (see reviews in [14, 28, 32]). However, if seroprevalence provides evidence of past exposure, it does not inform about the timing or frequency of infection. These latter are usually explored by estimating incidence rates [5]. To our knowledge, only [35, 36] have focused on estimating incidence rates in domestic cat populations, but none of these studies focused on farm cats.

In this study, the determinants of *T. gondii* seroconversion dynamics (used as a proxy of primo-infection dynamics) were investigated in five farm cat populations based on data from a two-year survey. Blood samples were collected and tested every three months to detect specific antibodies against *T. gondii*. Seroconversion rates were estimated in relation to the cats’ age and gender and to the season using multi-event capture-mark-recapture (CMR) to account for the uncertain age and serological status of cats that were not captured and not tested for *T. gondii* antibodies. The probabilities of misclassifications in the assignation of serological status (i.e. apparent false-negative and false-positive results) was also estimated since multi-event CMR models do not require a one-to-one correspondence between a test result and the serological status, but rather estimate the probabilities of each test results conditional on each serological status (e.g. a seropositive cat may be tested as having no *T. gondii* antibodies but with a different - much lower - probability than a seronegative cat). Although these values do not indicate the intrinsic bias due to the performance of diagnostic test, they allow to examine the apparent misclassification bias and its related factors.

## Methods

### Study site and cat populations

**Fig. 1:**
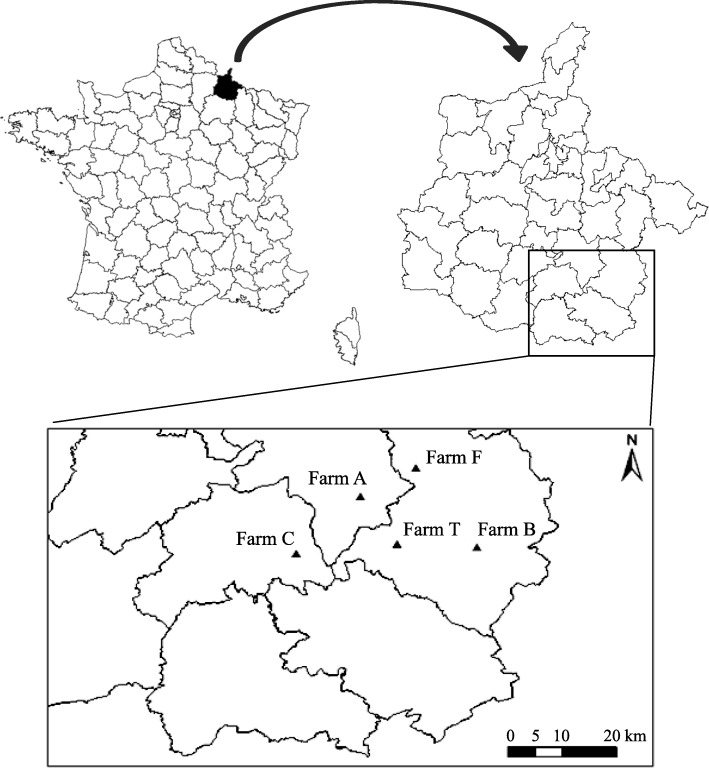
Location of the five study sites in the Ardennes region in France

The study was conducted on five populations of domestic cats living on five dairy farms (farms A, B, C, F and T) located in five villages in the Ardennes region of north eastern France (49°27'3.49"N, 4°47'0.7"E to 49°28'09"N, 5°00'52"E; Fig. 1). A recent study conducted on these farms found the soil to be highly contaminated with *T. gondii* [31], let expecting a high incidence of *T. gondii* in host populations. The five studied cat populations were considered as independent units since the sampled farms were between 3.4 and 14 km distant from each other, while the range of domestic cat movement around farms in the study area has been shown not to exceed 2.5 km [37]. The studied cats were free-ranging and mostly dependent on predation to survive as they were not fed or barely fed by farm owners. Their reproduction was not controlled, except on farm A where the first litters of the reproductive period (in spring) were sometimes removed by the farm owner.

### Cat capture and anaesthesia

The five cat populations were surveyed from April 2014 to January 2016. Captures were performed four times per year: in January (winter), April (spring), July (summer) and October (autumn) for a total of eight sampling sessions. On each sampling session, 8–10 cage traps (100 × 30 × 30 cm) were baited with dry cat food and distributed over the farmyard for a period of 2–4 days. The number of traps and the duration of the trapping session per farm were determined on the first session, depending on the specific requirements, in order to capture all the individuals in the population (visually observed and/or reported by farm owners). After the first session, these parameters remained unchanged for a given population to ensure similar capture effort over the study period. Cage traps were mainly used for unsociable cats that could not be approached, while sociable cats that could be approached by humans were caught by hand. On each session, captured cats were anaesthetised directly on the farm with isoflurane gas in order to take a blood sample. All cats were individually identified by the colour pattern of their coat or by a coloured plastic collar when having a uniform coat colour. They were also permanently marked with passive integrated transponder (PIT) tags implanted subcutaneously when first anaesthetised. Kittens less than two months old and cats in bad health (e.g. presenting symptoms of upper respiratory illness or extremely thin) were released without being tested. For each captured cat, the age class and gender were recorded. Three age classes were considered: ‘kittens’, from two to three months-old; ‘juveniles’, from four to six months-old; and ‘adults’, over 6 months-old. The age class was estimated based on birth observation or other information collected from farm owners and/or on teeth development [38]. Cat sociability towards humans was also recorded: ‘easy to catch by hand’ or ‘need trap to be caught’. Lastly, information on cat mortality events was also collected from farm owners to complement the dataset.

### Assessing cat immunity against *Toxoplasma gondii*

On each capture, a blood sample was taken from the marginal ear vein of the cat using a sterile needle. The drops of blood were applied on blotting paper (Whatman 3MM CHR) until a 2–3 cm^2^ surface area was saturated with blood. The blotting paper was then placed in a closed drying box with individual compartments and stored overnight at ambient temperature. When the blood had completely dried, blotting papers were placed in an individual envelope, labelled with the animal’s number and the collection date, and stored at -20 °C for a maximum of one month before performing serological tests. For these tests, a 1 × 1 cm strip of blotting paper was cut out using clean scissors, eluted in 300 μl of phosphate-buffered saline solution (PBS, pH 7.2) and incubated while being agitated overnight. After centrifugation, the supernatant (i.e. the eluate) was collected and tested for the presence of IgG antibodies to *T. gondii* using the modified agglutination test (MAT). Results were recorded as titres, corresponding to the inverse of the highest dilution for which an agglutination reaction was observed. All serological analyses were performed by the same person to avoid bias in agglutination detection.

Based on experimental results on cats [33, 39, 40], most epidemiological studies have used a cut-off value of 20 or 25 to discriminate between negative and positive results obtained from MAT tests (see reviews in [28, 32]). However, false negatives based on the cut-off value have been observed in experimentally infected cats even though the parasite was still present in their tissues [34]. Furthermore, the elution step performed for serological tests on blotting paper can weakly dilute antibody quantities, which may result in antibody titres inferior to those normally obtained with a standard serum [41, 42] and a misclassification in the assignment of serological status, in particular, producing false-negative results (titre < 25 from a seropositive individual). As multi-event models allow a serological status to give rise to any titre but with different probabilities [6], three titre classes were considered in the models: ‘titre = 0’ (most likely with seronegative individuals), ‘titre ≥ 25’ (most likely with seropositive individuals) and ‘0 < titre < 25’ (intermediate titres).

### Seroprevalence estimation

As per [35, 36], *T. gondii* seroprevalence in the five cat populations was estimated over the study period by randomly selecting one antibody measurement for each cat. The proportion of “seropositive” cats and the associated Wilson’s confidence intervals at 95% were calculated considering two cut-off values: (i) titre = 25 (the value generally used with serum samples); and (ii) titre = 10 (one titre lower than the value generally used with serum samples). In order to simulate a large number of the possible combinations using one measurement per cat, this calculation was repeated 1000 times. Overall seroprevalence in a population was then estimated as the mean proportion of seropositive cats in the 1000 re-samplings.

### Multi-event capture-mark-recapture modelling

Because unsociable cats were not captured during some sampling sessions, we performed analyses under the general framework of multi-event capture-mark-recapture models (CMR) accounting for uncertainties about state of some individuals (*sensu* ‘partial observations’) and for heterogeneity in capture rates between sociable and unsociable cats. Two sources of uncertainty in state assessment were observed in the dataset and considered in the models: (i) uncertainty about the serological status of some re-sighted individuals whose blood was not collected and thus not tested for the presence of *T. gondii* antibodies; and (ii) uncertainty about the age class of some individuals due to incomplete information about birth date and teeth. These uncertainties were considered explicitly in the capture histories by taking into account 15 events defined as follows: 0, not detected; 1, kitten tested with titre = 0; 2, kitten tested with titre ≥ 25; 3, kitten detected but not tested; 4, juvenile tested with titre = 0; 5, juvenile tested with titre ≥ 25; 6, juvenile detected but not tested; 7, adult tested with titre = 0; 8, adult tested with 0 < titre < 25; 9, adult tested with titre ≥ 25; 10, adult detected but not tested; 11, individual of undetermined age tested with titre = 0; 12, individual of undetermined age tested with titre ≥ 25; 13, individual of undetermined age detected but not tested;14, cat recovered dead.

The events ‘kitten/juveniles/undetermined age tested with 0 < titre < 25’ were not considered since uncertainty about age class only concerned the assignment of kittens or juveniles and both of these classes presented titre > 25 when ≠ 0. The event ‘0, not detected’ could correspond to a living cat present on the farm but not observed, a living cat absent from the farm or a dead undetected cat.

The 15 events might correspond to six biological states: ‘seronegative kitten’, ‘seropositive kitten’, ‘seronegative juvenile’, ‘seropositive juvenile’, ‘seronegative adult’ or ‘seropositive adult’. In addition, as information about dead cat recoveries was available from farm owners, the states ‘newly dead’ and ‘dead’ were also considered.

### Multi-event design

**Fig. 2:**
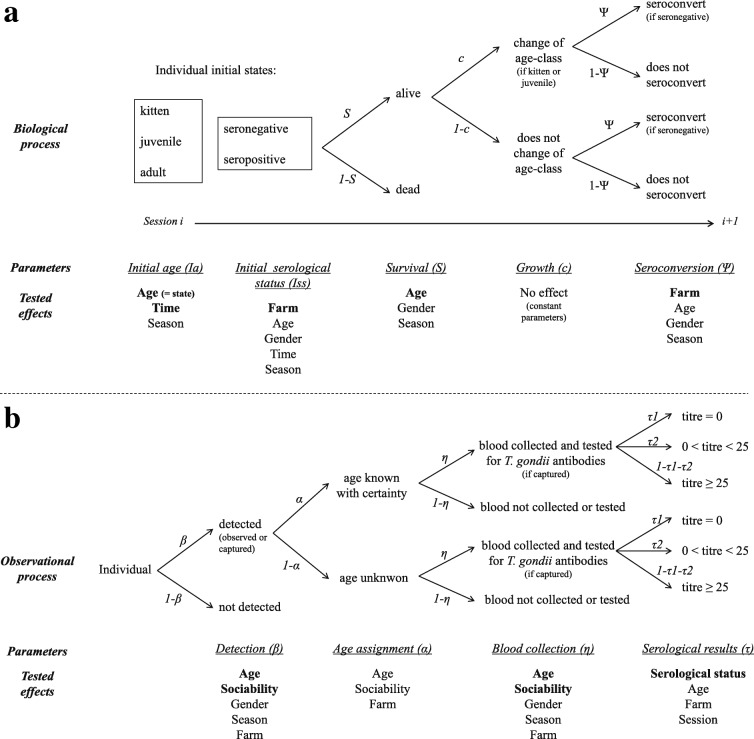
Diagrams of the biological process (**a**) and the observational process (**b**) used in multi-event CMR models. The diagram **a** describes the initial states and the conditional transitions between states from one sampling session to the next and the diagram **b** describes the recorded events for each cat at a given sampling session. The names of parameters corresponding to transitions and events and the effects tested on these parameters are indicated under each step of the processes. Effects written in bold correspond to the effects considered in the initial model. The detailed matrices used to estimate each parameter are presented in Additional file 1

The unobserved biological process in CMR models is described by *initial state probability* (*I*), which is the estimated probability of an individual being in a specific state when first captured, and *conditional transition probability* (*Φ*), which is the estimated probability of an individual moving from one state to another between two successive sessions following its first capture (Fig. 2a). We decomposed initial states in two parameters: (i) *initial age class* (*Ia*), corresponding to the probability of an individual belonging to one of the three age classes when first captured; and (ii) *initial serological status* (*Iss*), corresponding to the probability of an individual being seronegative or seropositive when first captured (Fig. 2a and see Additional file 1 for details regarding the matrices constructed for parameters estimation). Conditional transitions were broken down into three steps corresponding to three parameters: (i) *survival* (*S*), the estimated probability of an individual surviving between two successive sessions; (ii) *growth* (*c*), the estimated probability of an individual moving to the next age class between two successive sessions (conditional on its being alive); and (iii) *seroconversion* (*Ψ*), the estimated probability of an individual moving from the state ‘seronegative’ to the state ‘seropositive’ between two successive sessions, i.e. during a three months period (Fig. 2a). The three cat age classes (2–3 months, 4–6 months, and > 6 months) were chosen in such a way that an individual recorded in a given age class at session i systematically moved to the next age class at session i+1 (which occurred three months after session i), except for adult cats which remain in the same age class. Consequently, growth rates were considered constant and fixed to 1 (*c* = 1). Furthermore, as *T. gondii* antibodies have lifetime persistence in domestic cats [33], the transition probability from ‘seropositive’ to ‘seronegative’ was fixed at zero (see Additional file 1 for details regarding the matrices constructed for parameters estimation).

Event probabilities conditional on underlying states (*B*) were broken down into four steps (Fig. 2b and see Additional file 1 for details regarding the matrices constructed for parameters estimation): *detection* (*β*), the estimated probability of an individual being detected (i.e. observed or captured) at a given session; *age assignment* (*α*), the estimated probability of an individual being correctly assigned to a given age class at a given session when detected; *blood collection* (*η*), the estimated probability of collecting a blood sample from an individual and testing it for the presence of *T. gondii* antibodies (conditional on being detected); and *serological result* (*τ*), the estimated probability of an individual being assigned to a given class of titre values (conditional on blood collection and testing). This last parameter especially allows to estimate the probability a cat assigned as seropositive by the model shows null or low antibody titres (i.e. apparent false negative results) and the probability a cat assigned seronegative by the model shows positive antibody titres (i.e. apparent false positive results).

### Tested variables

Cat gender (*gender*) and sociability towards humans (*soc*) were integrated in the models as individual covariates. The gender of 23 kittens that died prematurely before being captured was randomly attributed based on a sex ratio at birth of 1:1 [43]. It has been previously reported that survival in free-ranging cats varies according to gender [44]. The variable *gender* was then tested on survival, as well as on detection and blood collection rates since sexual behaviour may affect the probability of cat presence on farms depending on the season [45]. Gender was also tested on initial serological status and seroconversion rates, although only a few studies have found a consistent relationship between gender and *T. gondii* seroprevalence in cat populations [46–48]. As the prevalence of *T. gondii* infection in cats and oocyst excretion vary according to season [49–51], this effect (*season*) was tested on seroconversion rates, as well as on survival, detection and blood collection rates. In addition, a group effect of the farm (*farm*) was also tested on almost all the parameters. This factor should reflect the effects of the farm’s environment, management and cat population characteristics known to influence the infection risk for animals on farms [52]. Finally, as the quantity of blood collected on the blotting papers was increased after the third sampling session, thanks to acquired technical skills, and potentially resulting in an increase in MAT accuracy to detect *T. gondii* antibodies on blotting papers, an effect of the period (before the third session *versus* after the third session) was tested on the serological results obtained. All the variables tested on the estimated parameters are summarised in Fig. 2.

### Goodness-of-fit tests and model selection

No specific test exists to detect a structural lack of fit of multi-event models to data [9]. In this study, the goodness-of-fit of the multi-event model was assessed after pooling all ‘detected alive’ events, resulting in a multi-state dataset containing only alive and dead encounters and ignoring uncertain states. The goodness-of-fit for the fully time-dependent Jolly-Move (JMV) multi-state model [53] was then applied using the U-CARE program version 2.3.2 [54].

Multi-event models were fitted using the E-SURGE program version 1.9.0 [55]. The model selection procedure was performed following a bottom-up approach (see Additional file 1: Table S1 for details on the starting model and the selection procedure). First, the best structure for initial states, i.e. detection, age assignment, blood sample and survival was selected. The last selection was conducted for serological results and seroconversion rates as these were the parameters of primary interest. In a final check, all models neighbouring the model with the best fit were examined. The relative support of competing models was assessed using the Akaike information criterion corrected for small sample size and possible overdispersion (QAICc) [56]. Models with the lowest QAICc were retained as good candidates, based on the criteria that a model fits the data significantly better when having two QAICc-points less than competing models [56]. If the difference in QAICc between models was less than two, the one with the smallest number of parameters was retained.

### Model assumptions and starting model structure

Rather than considering all possible models, only those related to *a priori* biological hypotheses were specified and compared. The following assumptions were used to build the starting model: (i) *initial age* (*Ia*) structure varies according to time *t* (i.e. sampling session); a higher proportion of young individuals in a population was expected from sessions performed during the cat breeding season (March-October) [45, 57]; (ii) *initial serological status* (*Iss*) and *seroconversion rates* (*Ψ*) vary between farms due to differences in soil contamination with *T. gondii* [31]; (iii) *survival rates* (S) vary according to cat age class; generally being lower for kittens than for juveniles and adults [44, 58]; (iv) *survival rates* (S) are considered equal for seronegative and seropositive individuals since *T. gondii* infection rarely induces acute toxoplasmosis and death in domestic cats [32]; (v) *detection* (*β*) and *blood collection* (*η*) *rates* are not affected by the serological status (*ss*) of individuals but vary with age, since kittens and juveniles are more systematically detected and caught for blood analysis and sociability towards humans since cats caught by hand can be detected and submitted to blood analysis at each session, contrary to unsociable cats; and (vi) *serological results* (*τ*) vary according to the inferred serological status (*ss*); we considered a seropositive cat has a greater probability to show positive antibody titres than a seronegative one and that a seronegative cat has a greater probability to have no antibody than a seropositive one. Based on these assumptions, the starting model had the following structure: *Ia*(*t*), *Iss*(*farm*), *S*(*age*), c, *Ψ*(*farm+soc*_*ad*_), *β*(*age*), *α*, *η*(*age+soc*_*ad*_), *τ*(*ss*), where parentheses indicate an effect of the variables on the parameter and *soc*_*ad*_ is the effect of sociability on adults only.

## Results

### Cat populations

The size of the cat populations varied between 7–25 individuals per farm depending on the period (i.e*.* kitten season or not). The average number of cats (± SD) observed per farm and per session varied from 10.37 ± 3.25 to 21.62 ± 4.41 (Table 1). A total of 154 cats were detected (observed and/or captured) during the study period. Of these cats, 130 (84.41%) were tested at least once for the presence of *T. gondii*-specific antibodies. The remaining 24 individuals were never tested because they were kittens that died before weaning. In total, 400 serological tests were carried out (one to eight per individual; see Table 1 for details). During the study period, 91 cats were born: 32 on farm A, 22 on farm B, 16 on farm C, 17 on farm F and 4 on farm T. The age was known to the nearest week for 79 of these individuals (86.81%). The 12 cats for which it was impossible to assign an age class with certainty represented 13.19% of the cats born during the study and 7.79% of the total cats.

**Table 1:** Average number of cats observed per farm and per sampling session and number of cats tested for the presence of *T. gondii* antibodies at one to eight times

Farm	Average number of cats observed per sampling session (± SD)	Number of cats tested								
		Once	Twice	3 times	4 times	5 times	6 times	7 times	8 times	At least once
A	21.62 ± 4.41	10	11	6	3	8	2	1	0	41
B	16.33 ± 5.24	8	10	5	4	0	0	0	0	27
C	14.37 ± 3.96	6	3	4	1	0	2	7	0	23
F	19.62 ± 3.78	10	4	3	2	2	3	2	0	26
T	10.37 ± 3.25	4	2	0	0	2	1	2	2	13
Total	73.75 ± 12.02	38	30	18	10	12	8	12	2	130

*Abbreviation*: *SD* standard deviation

### Observed antibody titres from the MAT

Transitions from ‘titre = 0’ to ‘titre ≠ 0’ (i.e. intermediate titre or titre ≥ 25) were observed for 28 individuals (see Additional file 2: Table S3 for details of the data): 15 young cats born during the study (for which post-transition titres varied from 25 to 12,800) and 13 adult cats (for which post-transition titres varied from 10 to 2500). Of the 91 cats born during the study, 15 (16.48%) showed titres ≥ 25 at the first capture. Of these 15 cats, two were less than three months and showed antibody titres of 400 and 1600, respectively, nine were 4–6 months old, three could not be assigned in an age class and one was adult. Eleven of these cats originated from farm A (34.37% of kittens born during the study on this farm) and four from farm F (23.53% of the kittens born during the study on this farm).

Based on a cut-off value of titre = 25, the estimated seroprevalence over the study period varied from 15.38% in farm T to 73.08% in farm F (see Table 2). When a cut-off value of titre = 10 was considered, seroprevalence varied from 29.63% in farm B to 73.08% in farm F (Table 2).

**Table 2:** Seroprevalence, annual seroconversion rates and the observed range of antibody titres per farm for the full study period. Annual seroconversion rates were calculated from the quarterly seroconversion rates estimated by the retained multi-event CMR model

Farm	Seroprevalence (95% CI) (%)		Annual seroconversion rate	Antibody titres (≠ 0)
	With a cut-off value of 25	With a cut-off value of 10		
A	65.85 (50.55–78.44)	70.73 (55.52–82.39)	0.96	6–12,800
B	29.63 (15.85–48.48)	29.63 (15.85–48.48)	0.42	10–6400
C	34.78 (18.81–55.11)	52.17 (32.96–70.76)	0.88	6–400
F	73.08 (53.92–86.30)	73.08 (53.92–86.30)	0.96	10–12,800
T	15.38 (94.33–42.23)	38.46 (17.71–64.48)	0.42	10–100

*Abbreviation*: *CI* confidence interval

### Goodness-of-fit tests

The fully time-dependent Jolly-Move model, which ignores uncertainty and serological status, did not fit the data (*χ*^2^ = 36.04, *df* = 21, *P* = 0.02). Based on the details of the tests (test 3G.Sm: *χ*^2^ =19.67, *df* = 6, *P* = 0.003), we suspected that a major cause of lack of fit could be a rather strong heterogeneity of detection among individuals. When sociability towards humans (‘caught only with traps’ *versus* ‘caught by hand’) was taken into account, there remained no evidence of structural failure in the application of the fully time-dependent Jolly-Move model (*χ*^2^ = 21.19, *df* = 19, *P* = 0.33 and *χ*^2^ = 13.77, *df* = 9, *P* = 0.13 for group 1 and 2, respectively). An effect of sociability on detection rate was thus considered in the starting model. Cat age was also a variable which may favour the detection of heterogeneity in our dataset, since cats ≤ 6 months were almost systematically detected at each occasion contrary to adults. This effect was taken into account in the models and tested on each parameter.

### Seroconversion rates estimated by multi-event CMR models

**Fig. 3:**
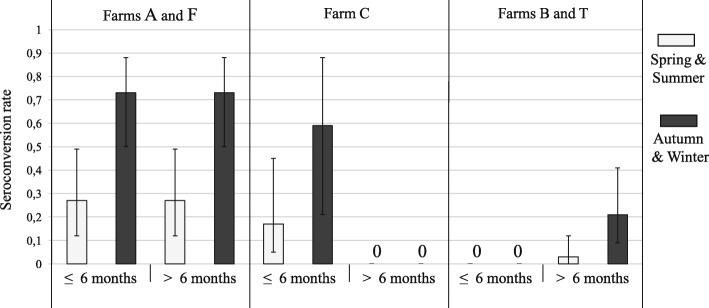
Seroconversion rates estimated by the best retained model. The model considers the interactive effects of cats age (≤ 6 months and > 6 months) and population (farms A, B, C, F and T) and an additive effect of season (Spring, Summer, Autumn and Winter). Error bars represent standard errors

To investigate seroconversion rates, 95 competing models were performed (Additional file 1: Table S1). In the most supported model, estimated quarterly seroconversion rates (*Ψ*) varied according to age class, farm and season (Table 3 and Additional file 1: Table S1). A general seasonal pattern was observed on all the farms: quarterly seroconversion rates were higher from October to April than from April to October (Fig. 3). Seroconversion rates and their distribution among age classes also varied according to farm (Fig. 3 and Additional file 1: Table S2 for details of the estimated parameters). These rates were higher on farms A and F than on the other farms and did not differ between age classes (*Ψ* = 0.27 for the periods April-July and July-October, and *Ψ* = 0.73 for the periods October-January and January-April). Lower seroconversion rates were observed on farm C and occurred only in cats ≤ 6 months (*Ψ* = 0.17 and *Ψ* = 0.59). Seroconversion rates were lowest on farms B and T and occurred only in adult cats (*Ψ* = 0.04 and *Ψ* = 0.21). Based on these parameters, the annual seroconversion rates were estimated based on the following formula: *Ψ*_annual_ = 1 - [(1 - *Ψ*_January-April_) (1 - *Ψ*_April-July_) (1 - *Ψ*_July-October_) (1 - *Ψ*_October-January_)] and were equal to 0.42 seroconversions/cat/year on farms B and T, 0.88 seroconversions/cat/year on farm C and 0.96 seroconversions/cat/year on farms A and F (Table 2).

**Table 3:** Summary of multi-event selection for the estimation of seroconversion rates (*Ψ*) and serological results (*τ*)

Parameters	Model parameterization^a^	Dev	K	QAICc	∆QAICc
Seroconversion (*Ψ*)	[farm (A & F) + age (K & J, Ad) . farm (B & T, C)] + season (Sp & Su, Au & W)	1971.9	51	2082.9	0.0
	Farm (A & F) + age (K & J, Ad) . farm (B & T, C)	1981.6	51	2092.6	9.7
	Farm (A & F, B & T, C) + season (Sp & Su, Au & W)	1984.7	50	2093.3	10.4
	Age (K & J, Ad) + season (Sp & Su, Au & W)	2007.7	49	2113.9	31.0
Serological result (*τ*)	[ssN + ssP . farm (A & B & F, C & T)] + *t* (1 & 2 & 3, 4 & 5 & 6 & 7 & 8)	1971.9	51	2082.9	0.0
	ssN + ssP . farm (A & B & F, C & T)	1985.9	51	2096.9	14.0
	[ssN + ssP] + t (1 & 2 & 3, 4 & 5 & 6 & 7 & 8)	1993.1	50	2101.7	18.8

^a^The structure of the other parameters (initial states, *survival*, *detection*, *blood collection* and *age assignment*) is the same for all seven models listed in this table (see Additional file 1: Table S1 for the details of the model parameterization for these parameters). The ‘.’ entries (‘dot’) denote an interactive effect, ‘+’ entries denote an additive effect, and ‘&’ entries specify that two or more parameters are equal. Square brackets indicate that the additive effect is applied to all the terms contained in the square brackets. All other competing models, representing various structures for the other parameters, are presented in Additional file 1: Table S1. None of the competing models were supported by the data (ΔAICc > 2)

*Abbreviations*: *A* farm A, *B* farm B, *C* farm C, *F* farm F, *T* farm T, *K* kitten, *J* juvenile, *Ad* adult, *Sp* spring, *Su* summer, *Au* autumn, *W* winter, *ssN* seronegative, *ssP* seropositive, *t* sampling session time

### Distribution and estimated occurrence probabilities of antibody titre classes

**Fig. 4:**
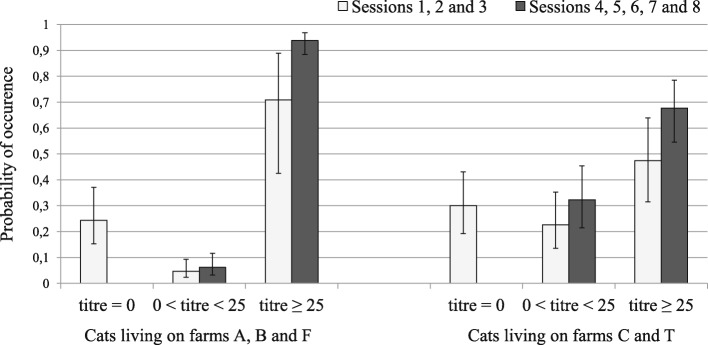
Occurrence probabilities of antibody titre classes in cats assigned as seropositive according to the best retained model. The model considers the effect of farms and sampling sessions. The white bars represent sessions before methodological improvements in sampling, and the black bars represent sessions after these improvements. Error bars represent standard error

The distribution of antibody titres ≠ 0 differed between cat populations. On farms C and T, most cats showing titres ≠ 0 exhibited titres between 10 and 100, whereas on farms A, B and F, titres were most often > 100 (see Table 2 and Additional file 2: Table S3 for details of the data). Similar results were also observed in the retained model (Table 3), in which cats assigned as seropositive by the model and living on farms C and T, had a probability of showing intermediate titres (i.e. 0 < titres < 25) of *τ* = 0.23 and *τ* = 0.32, and a probability of showing titres ≥ 25 of *τ* = 0.47 and *τ* = 0.68, during sessions 1–3 and sessions 4–8, respectively. In contrast, cats assigned as seropositive by the model and living on farms A, B and F, had a probability of showing intermediate titres almost null (*τ* = 0.05 and *τ* = 0.06), whereas they had a probability of showing titres ≥ 25 of *τ* = 0.71 and *τ* = 0.94, in sessions 1–3 and sessions 4–8, respectively (Fig. 4).

In addition, according to the selected model, antibody titres ≥ 25 or intermediate titres rarely occurred in cats assigned as seronegative by the model. The estimated frequencies of these titres did not reach 0.04 (Additional file 1: Table S2). In contrast, titres = 0 were frequently observed in cats assigned as seropositive by the model at the beginning of the study (*τ* = 0.24 to 0.30 in sessions 1–3) whereas no titres = 0 were detected after the third session when the quantity of blood collected was increased (i.e. sessions 4–8).

## Discussion

To our knowledge, this study provides for the very first time a detailed description of intra-annual *T. gondii* seroconversion dynamics in cats living on farms.

### Seroprevalences and seroconversion rates among farm cat populations

The average annual seroconversion rates estimated on the five studied farms (0.42 for the farms B and T, 0.88 for the farm C and 0.96 for the farms A and F) are high compared to those previously reported in an urban population of stray cats (0.17 seroconversions/year) [35] and in three rural populations of owned domestic cats (0.26, 0.36 and 0.39 seroconversions/year) [36]. These results support previous findings showing that livestock farms are hotspots of *T. gondii* infection in rural areas [29, 31, 59–61]. However, the seroconversion rates, as well as the estimated seroprevalences were highly variable between farms (from 15.38 to 73.08% or from 29.63 to 73.08% depending on the selected cut-off value) and the range of seroprevalences we obtained corresponds to the range of seroprevalences in farm cats previously reported in the literature: 33.3% in Spain [62]; 41.9% [63]; 68% [64] and 53.19% [65] in the USA; 62% in the UK [66]; and 75% in Poland [67]. The spatial heterogeneity we observed in our study could result from variations in many factors such as: (i) precipitation [35, 36]; (ii) the number of surrounding farms [29]; (iii) prey availability and access to food [68]; and/or (iv) the number of kittens in the cat population [36].

### Seasonal variations of seroconversion rates

Seroconversion rates in farm cats vary temporally according to the season, with significantly higher rates during autumn and winter (from 0.21 to 0.73 between October and April) than during spring and summer (from 0.04 to 0.27 between April and October). These variations are in line with the predictions of [69] suggesting that cat seroprevalence and oocyst excretion are the result of two non-exclusive factors: (i) host population dynamics following intra-annual cycles; and (ii) oocyst survival depending on seasonal climatic variations. Seroconversion probability in cats would be low in spring and summer because newborn kittens, which represent the most susceptible part of the population, are protected from infection by maternally derived antibodies [35, 70]. Furthermore, oocyst survival is expected to be lower during dry and hot periods [71, 72] corresponding to summer in the study area. In contrast, moisture and mild temperatures in autumn, combined with an increasing proportion of infected intermediate host prey (birds and small mammals), would favour the infection risk for cats [69].

In previous studies carried out in Germany [50, 51], the proportion of owned cat faeces with *T. gondii* oocysts was lower in winter (December to March) than during the rest of the year while in our study, seroconversion rates in farm cats were high between January and April. The latency between oocyst shedding and seroconversion in cats [32] may partly explain this difference but it could also originate from differences in prey availability between cat populations. A low availability of infected prey in winter could explained the low risk of infection for free-ranging owned cats in Germany [50, 51], while the concentration of commensal rodent species in farm buildings [30] could maintain a high risk of farm cats infection during winter in our study area. In addition, oocysts located inside barns may remain viable and infectious during winter since farms are considered to be moist, shady environments favourable to their survival [59].

### Age of infection

Considering the short time span of the survey and the high seroprevalences on farms, surprisingly high seroconversion rates were observed in adult cats on some farms (up to 0.59 on farm C and up to 0.73 in farms A and F). It cannot be excluded that these high seroconversion rates partly result from a previous decrease in immune response in some individuals as has been reported for experimentally infected cats with low *T. gondii* antibody titres [34]. However, apparent seroconversions may also arise from false-negative results obtained with MAT, especially for the three first sampling sessions when the probability to observe titres = 0 in cats assigned as seropositive by the model were 0.24 to 0.30. As a consequence, some of the transitions from negative to positive titres occurring in adult cats may have been erroneously considered as seroconversions in the models, resulting in an overestimation of the seroconversion rate in cat populations.

In contrast, specific *T. gondii* antibodies were detected in very young individuals: two kittens aged 2–3 months and one young cat of around three months (assigned in the uncertain age state). Seropositivity in these kittens may result from maternal antibodies which are passively transferred from the colostrum and that can be detected in kitten serum until the age of 10–12 weeks [46, 70]. However, it may also result from an actively acquired infection since titres in the three cats were high (1600, 400 and 3200, respectively) and increased or remained high (3200 for the three individuals) three months later. As a mother starts bringing her kittens dead prey when they are about four weeks of age [73], kittens can become infected through the consumption of tissue cysts from the end of the weaning period (after eight weeks). For example, in a natural population of stray cats, [46] reported that five kittens aged 5–9 weeks had acquired *T. gondii* infection.

### Effect of young cats on infection risk in farms

Despite the potential bias reported above (“false” seroconversions in adults and presence of maternal derived antibodies in kittens), different age patterns of seroconversion were observed between farms. These patterns seem to depend on the infection risk on the farm since cats can get infected before reaching six months of age on farms A, F and C, where seroconversion rates and seroprevalence are high, whereas infections only occurred in adults on farms B and T where seroconversion rates and seroprevalences are lower. Infections in young farm cats have been previously reported based on the detection of both *T. gondii*-specific antibodies [64] and *T. gondii*-like oocysts in faeces [74]. The renewal of individuals in farm cat populations is generally high since their reproduction is not, or poorly, controlled by farm owners. This results in a high proportion of susceptible new individuals each year. Only some of these individuals survive to the point of getting infected, as kittens of 2–3 months in our study have an average survival rate of 0.57 (see the results for survival rates in Additional file 3). However, on farms where the risk of exposure to *T. gondii* is high, young cats can get infected early, so even if they die young they can contribute to environmental contamination by shedding oocysts. In this way, kittens may contribute to the maintenance of oocysts on farms with high *T. gondii* contamination. In this case, a control strategy of sterilising cats to limit the annual number of new susceptible cats should help reduce environmental contamination.

### Variability of serological titres

High heterogeneity in *T. gondii* antibody titres among seropositive cats has been observed both experimentally [33] and in natural cat populations [35]. Cats can exhibit four to eight-fold variations in maximal titres after ingesting the same number of bradyzoites [33]. Our study also found that the pattern of antibody titres varied according to the different cat populations, most of the cats on farms B and T showed low to medium titres (mostly from 10 to 100), whereas the cats on farms A, C and F showed high titres (> 100, up to 12,800). As noted in other studies, these variations in antibody titres may be explained both by host factors, such as genotypes (as observed in mice [75]), health status and/or coinfection with other parasites and virus [76–78] and/or by the strain of *T. gondii* that may impact the level of produced antibodies in cats [33, 39]. However, further studies are needed to understand how immune response varies from a cat to another and at a larger range from a cat population to another.

## Conclusion

Based on longitudinal serological data collected in five cat populations every three months over a two-year period, variations in seroconversion rates were observed between farms but also between seasons, suggesting that risks of exposure to *T. gondii* oocysts for humans and livestock differ during the year. Thanks to the high frequency of blood sampling, our study allowed the detection of seroconversion occurring early in young cats on farms where parasite exposure seems to be greatest. It suggests that kittens, often present in a large number on a farm, may be responsible for contributing to a significant part of oocyst shedding and thus for maintaining high levels of environmental contamination by *T. gondii*. These results provide important insight for understanding *T. gondii* infection dynamic in natural cat populations. In addition to these biological findings, this study highlights the utility of multi-event CMR modelling approaches in order to estimate seroconversion rates from partial serological data collected on untamed outdoor cat populations, and the apparent risk of misclassification in serological status based on serological tests.

## Additional files

Additional file 1: **Text.** Matrices constructed for parameters estimation in the E-SURGE program. **Table S1.** Detailed structure of the set of models and the model selection procedure from the multi-event analysis. **Table S2.** Estimated parameters from the selected model. (DOCX 53 kb)

Additional file 2: **Table S3.** Dataset showing Toxoplasma gondii antibody titres obtained from the modified agglutinated test (MAT) and the characteristics of the sampled cat. (DOCX 51 kb)

Additional file 3: Description of survival rates, detection rates, age assignment and blood collection rates estimated from the best retained model. (DOCX 12 kb)

## Abbreviations

CMR: Capture-mark-recapture
MAT: Modified agglutination test
