# Supplementary material for: A multi-event capture-recapture analysis of Toxoplasma gondii seroconversion dynamics in farm cats
Source: Parasit Vectors. 2018 Jun 8;11:339. doi: 10.1186/s13071-018-2834-4 (PMC5994099; doi:10.1186/s13071-018-2834-4)
Supplement: Supplementary file 1 — Text. Matrices constructed for parameters estimation in the E-SURGE program. Table S1. Detailed structure of the set of models and the model selection procedure from the multi-event analysis. Table S2. Estimated parameters from the selected model. (DOCX 53 kb) [file 13071_2018_2834_MOESM1_ESM.docx]

**Additional file 1: Detailed structure of the set of models used in the multi-event analysis**

This file presents the matrices constructed for parameters estimation in the E-SURGE program, the model selection procedure from the multi-event analysis and the estimated parameters from the selected model.

The multi-event models used in this analysis were implemented in the E-SURGE program (Choquet *et al*., 2009). The models were based on nine parameters: two initial state probabilities; three state transition probabilities; and four event probabilities. The basic structure, shared by all the models, was specified with ‘pattern’ matrices in the ‘GEPAT’ module of E-SURGE. Here, ‘$*$’ entries denote the complement of the row-sum of entries (letters) and ‘$-$’ entries denote zeros (*i.e.* impossible combinations).

Initial age (*Ia*):

$\left[ \begin{matrix} * & i1 & i2 \end{matrix} \right]$

Initial serological status (*Iss*):

$\left[ \begin{matrix} * & \pi1 & - & - & - & - & - & - \\ - & - & * & \pi2 & - & - & - & - \\ - & - & - & - & * & \pi3 & - & - \end{matrix} \right]$

Survival (*S*):

$\left[ \begin{matrix} S1 & - & - & - & - & - & * & - \\ - & S1 & - & - & - & - & * & - \\ - & - & S2 & - & - & - & * & - \\ - & - & - & S2 & - & - & * & - \\ - & - & - & - & S3 & - & * & - \\ - & - & - & - & - & S4 & * & - \\ - & - & - & - & - & - & - & * \\ - & - & - & - & - & - & - & * \end{matrix} \right]$

Growth (*c*):

$\left[ \begin{matrix} - & - & * & - & - & - & - & - \\ - & - & - & * & - & - & - & - \\ - & - & - & - & * & - & - & - \\ - & - & - & - & - & * & - & - \\ - & - & - & - & * & - & - & - \\ - & - & - & - & - & * & - & - \\ - & - & - & - & - & - & * & - \\ - & - & - & - & - & - & - & * \end{matrix} \right]$

Seroconversion (*Ψ*):

$\left[ \begin{matrix} * & \Psi1 & - & - & - & - & - & - \\ - & * & - & - & - & - & - & - \\ - & - & * & \Psi2 & - & - & - & - \\ - & - & - & * & - & - & - & - \\ - & - & - & - & * & \Psi3 & - & - \\ - & - & - & - & - & * & - & - \\ - & - & - & - & - & - & * & - \\ - & - & - & - & - & - & - & * \end{matrix} \right]$

Detection (β):

$\left[ \begin{matrix} \beta1 & - & - & - & - & - & - & * \\ - & \beta1 & - & - & - & - & - & * \\ - & - & \beta1 & - & - & - & - & * \\ - & - & - & \beta1 & - & - & - & * \\ - & - & - & - & \beta2 & - & - & * \\ - & - & - & - & - & \beta2 & - & * \\ - & - & - & - & - & - & \beta3 & * \\ - & - & - & - & - & - & - & * \end{matrix} \right]$

Age assignment (α):

$\left[ \begin{matrix} \alpha1 & - & - & - & - & - & * & - & - & - \\ - & \alpha1 & - & - & - & - & - & * & - & - \\ - & - & \alpha2 & - & - & - & * & - & - & - \\ - & - & - & \alpha2 & - & - & - & * & - & - \\ - & - & - & - & \alpha3 & - & * & - & - & - \\ - & - & - & - & - & \alpha3 & - & * & - & - \\ - & - & - & - & - & - & - & - & * & - \\ - & - & - & - & - & - & - & - & - & * \end{matrix} \right]$

Blood sample (η):

$\left[ \begin{matrix} \eta1 & * & - & - & - & - & - & - & - & - & - & - & - & - & - & - & - & - \\ - & - & \eta1 & * & - & - & - & - & - & - & - & - & - & - & - & - & - & - \\ - & - & - & - & \eta2 & * & - & - & - & - & - & - & - & - & - & - & - & - \\ - & - & - & - & - & - & \eta2 & * & - & - & - & - & - & - & - & - & - & - \\ - & - & - & - & - & - & - & - & \eta3 & * & - & - & - & - & - & - & - & - \\ - & - & - & - & - & - & - & - & - & - & \eta3 & * & - & - & - & - & - & - \\ - & - & - & - & - & - & - & - & - & - & - & - & \eta4 & * & - & - & - & - \\ - & - & - & - & - & - & - & - & - & - & * & - & - & - & \eta4 & * & - & - \\ - & - & - & - & - & - & - & - & - & - & - & - & - & - & - & - & * & - \\ - & - & - & - & - & - & - & - & - & - & - & - & - & - & - & - & - & * \end{matrix} \right]$

Serological results (*τ*):

$$\left[ \begin{matrix} - & * & - & - & - & - & - & - & - & - & - & - & - & - & - \\ - & - & - & * & - & - & - & - & - & - & - & - & - & - & - \\ - & - & * & - & - & - & - & - & - & - & - & - & - & - & - \\ - & - & - & * & - & - & - & - & - & - & - & - & - & - & - \\ - & - & - & - & * & - & - & - & - & - & - & - & - & - & - \\ - & - & - & - & - & - & * & - & - & - & - & - & - & - & - \\ - & - & - & - & - & * & - & - & - & - & - & - & - & - & - \\ - & - & - & - & - & - & * & - & - & - & - & - & - & - & - \\ - & - & - & - & - & - & - & \tau1 & \tau2 & * & - & - & - & - & - \\ - & - & - & - & - & - & - & - & - & - & * & - & - & - & - \\ - & - & - & - & - & - & - & * & \tau3 & \tau4 & - & - & - & - & - \\ - & - & - & - & - & - & - & - & - & - & * & - & - & - & - \\ - & - & - & - & - & - & - & - & - & - & - & * & - & - & - \\ - & - & - & - & - & - & - & - & - & - & - & - & - & * & - \\ - & - & - & - & - & - & - & - & - & - & - & - & * & - & - \\ - & - & - & - & - & - & - & - & - & - & - & - & - & * & - \\ - & - & - & - & - & - & - & - & - & - & - & - & - & - & * \\ * & - & - & - & - & - & - & - & - & - & - & - & - & - & - \end{matrix} \right]$$

The covariate structure (variation by age, serological status, farm, occasion, *etc*.) of each matrix was defined in the ‘GEMACO’ module of E-SURGE. The structure of all the models is provided in Table S1.

**Table S1.** Results of the model selection procedure from the multi-event analysis.

The selection of relevant covariates was conducted step by step for each type of parameter in the following order: initial age (*Ia*); initial serological status (*Iss*); conditional transitions, i.e. survival (*S*) and seroconversion (*Ψ*); and event parameters, i.e. detection (*β*), age assignment (*α*), blood collection (*η*), serological result (*τ*). The tested covariates were: age (K, kitten; J, juvenile; Ad, adult; U, undefined); season (Sp, spring; Su, summer; Au, autumn; W, winter); farm (A, B, C, F, T); sociability towards humans (socS, sociable; socU, unsociable); serological status (ssN, seronegative; ssP, seropositive) and occasion time (t). ‘R’ denotes a cat recovered dead. The ‘i’ entries (for ‘intercept’) denote a constant parameter, ‘.’ entries (‘dot’) denote an interactive effect, ‘+’ entries denote an additive effect, and ‘&’ entries specify that two or more parameters are equal. For the purposes of clarity, the structure presented in Table S1 is not the same as the structure defined in the ‘GEMACO’ module of E-SURGE, but details on implementing models in the GEMACO module are provided by Choquet (2007, 2008). The model in bold is the selected model, i.e. the one that significantly better fit the data according to the Akaike information criterion corrected for small sample size and possible overdispersion (QAICc).

| **Initial states** | | **Conditional transitions** | | **Events** | | | |  |  |  |  |
| --- | --- | --- | --- | --- | --- | --- | --- | --- | --- | --- | --- |
| **Initial age (Ia)** | **Initial serological status (Iss)** | **Survival**  **(S)** | **Seroconversion (Ψ)** | **Detection**  **(β)** | **Age assignment**  **(α)** | **Blood collection (η)** | **Serological result (τ)** | **Dev** | **QAICc** | **N** | **Δ**  **QAICc** |
| t | farm | age | farm | K&J+soc(Ad)+R | i | age+soc(Ad) | ss | 2219.3 | 2300.2 | 38 | 217.3 |
| season | farm | age | farm | K&J+soc(Ad)+R | i | age+soc(Ad) | ss | 2225.7 | 2297.6 | 34 | 214.7 |
| J.season(Sp,SuAu,W)+Ad.season(Sp,Su,AuW) | farm | age | farm | K&J+soc(Ad)+R | i | age+soc(Ad) | ss | 2234.3 | 2304.0 | 33 | 221.0 |
| season+farm | farm | age | farm | K&J+soc(Ad)+R | i | age+soc(Ad) | ss | 2222.2 | 2305.4 | 39 | 222.5 |
| season+farm(ACF,B,T) | farm | age | farm | K&J+soc(Ad)+R | i | age+soc(Ad) | ss | 2225.0 | 2303.7 | 37 | 220.7 |
| season | age.farm | age | farm | K&J+soc(Ad)+R | i | age+soc(Ad) | ss | 2195.3 | 2287.7 | 43 | 204.8 |
| season | (K&J,Ad).farm | age | farm | K&J+soc(Ad)+R | i | age+soc(Ad) | ss | 2195.9 | 2279.1 | 39 | 196.2 |
| season | farm+season | age | farm | K&J+soc(Ad)+R | i | age+soc(Ad) | ss | 2222.2 | 2300.8 | 37 | 217.9 |
| season | farm.season | age | farm | K&J+soc(Ad)+R | i | age+soc(Ad) | ss | 2214.0 | 2311.0 | 45 | 228.1 |
| season | farm+t(1, 2 3 4 5 7 8) | age | farm | K&J+soc(Ad)+R | i | age+soc(Ad) | ss | 2223.8 | 2298.0 | 35 | 215.0 |
| season | farm.t(1, 2 3 4 5 6 7 8) | age | farm | K&J+soc(Ad)+R | i | age+soc(Ad) | ss | 2220.4 | 2301.3 | 38 | 218.4 |
| season | farm+gender | age | farm | K&J+soc(Ad)+R | i | age+soc(Ad) | ss | 2224.4 | 2298.6 | 35 | 215.6 |
| season | (K&J,Ad).farm(A,BCF,T) | age | farm | K&J+soc(Ad)+R | i | age+soc(Ad) | ss | 2196.5 | 2270.6 | 35 | 187.7 |
| season | (K&J,Ad).farm(A,C,BF,T) | age | farm | K&J+soc(Ad)+R | i | age+soc(Ad) | ss | 2195.9 | 2274.6 | 37 | 191.7 |
| season | (K&J,Ad).farm(A,BCF,T) +t(1, 2 3 4 5 6 7 8) | age | farm | K&J+soc(Ad)+R | i | age+soc(Ad) | ss | 2194.3 | 2270.8 | 36 | 187.8 |
| season | (K&J,Ad).farm(A,BCF,T) +gender | age | farm | K&J+soc(Ad)+R | i | age+soc(Ad) | ss | 2196.1 | 2272.5 | 36 | 189.6 |
| season | (K&J,Ad).farm(A,BCF,T) | age | farm | K&J+soc(Ad)+R.farm(ACFT,B) | i | age+soc(Ad) | ss | 2170.1 | 2246.5 | 36 | 163.5 |
| season | (K&J,Ad).farm(A,BCF,T) | age | farm | K&J+socS(Ad)+socU(Ad).farm+R.farm(ACFT,B) | i | age+soc(Ad) | ss | 2157.8 | 2243.3 | 40 | 160.4 |
| season | (K&J,Ad).farm(A,BCF,T) | age | farm | K&J+socS(Ad)+socU(Ad).gender+R.farm(ACFT,B) | i | age+soc(Ad) | ss | 2170.0 | 2248.7 | 37 | 165.8 |
| season | (K&J,Ad).farm(A,BCF,T) | age | farm | K&J+socS(Ad)+socU(Ad).season+R.farm(ACFT,B) | i | age+soc(Ad) | ss | 2161.8 | 2245.0 | 39 | 162.1 |
| season | (K&J,Ad).farm(A,BCF,T) | age | farm | K&J+socS(Ad)+socU(Ad).farm+R | i | age+soc(Ad) | ss | 2184.6 | 2267.9 | 39 | 184.9 |
| season | (K&J,Ad).farm(A,BCF,T) | age | farm | K&J+socS(Ad)+socU(Ad).farm(A,B,CF,T)+R.farm(ACFT,B) | i | age+soc(Ad) | ss | 2157.9 | 2241.0 | 39 | 158.1 |
| season | (K&J,Ad).farm(A,BCF,T) | age | farm | K&J+socS(Ad)+socU(Ad).farm(AT,B,CF)+R.farm(ACFT,B) | i | age+soc(Ad) | ss | 2159.0 | 2239.9 | 38 | 157.0 |
| season | (K&J,Ad).farm(A,BCF,T) | age | farm | K&J+socS(Ad)+socU(Ad).season(SpAuW, Su)+R.farm(ACFT,B) | i | age+soc(Ad) | ss | 2162.1 | 2240.8 | 37 | 157.8 |
| season | (K&J,Ad).farm(A,BCF,T) | age | farm | K&J+socS(Ad)+socU(Ad).farm(ABCFT).season(SpAuW, Su)+R.farm(ACFT,B) | i | age+soc(Ad) | ss | 2139.7 | 2241.3 | 47 | 158.4 |
| season | (K&J,Ad).farm(A,BCF,T) | age | farm | K&J+socS(Ad)+socU(Ad).farm(ABCFT)+season(SpAuW, Su)+R.farm(ACFT,B) | i | age+soc(Ad) | ss | 2151.5 | 2239.2 | 41 | 156.3 |
| season | (K&J,Ad).farm(A,BCF,T) | age | farm | K&J+socS(Ad)+socU(Ad).farm(AT,B,CF)+R.farm(ACFT,B) | age | age+soc(Ad) | ss | 2128.7 | 2214.2 | 40 | 131.2 |
| season | (K&J,Ad).farm(A,BCF,T) | age | farm | K&J+socS(Ad)+socU(Ad).farm(AT,B,CF)+R.farm(ACFT,B) | farm | age+soc(Ad) | ss | 2137.6 | 2227.7 | 42 | 144.8 |
| season | (K&J,Ad).farm(A,BCF,T) | age | farm | K&J+socS(Ad)+socU(Ad).farm(AT,B,CF)+R.farm(ACFT,B) | age.farm | age+soc(Ad) | ss | 2108.2 | 2221.5 | 52 | 138.6 |
| season | (K&J,Ad).farm(A,BCF,T) | age | farm | K&J+socS(Ad)+socU(Ad).farm(AT,B,CF)+R.farm(ACFT,B) | age+farm | age+soc(Ad) | ss | 2115.1 | 2209.8 | 44 | 126.9 |
| season | (K&J,Ad).farm(A,BCF,T) | age | farm | K&J+socS(Ad)+socU(Ad).farm(AT,B,CF)+R.farm(ACFT,B) | [(K,J).farm]+Ad | age+soc(Ad) | ss | 2112.1 | 2211.3 | 46 | 128.4 |
| season | (K&J,Ad).farm(A,BCF,T) | age | farm | K&J+socS(Ad)+socU(Ad).farm(AT,B,CF)+R.farm(ACFT,B) | K+[J.farm]+Ad | age+soc(Ad) | ss | 2118.8 | 2211.1 | 43 | 128.2 |
| season | (K&J,Ad).farm(A,BCF,T) | age | farm | K&J+socS(Ad)+socU(Ad).farm(AT,B,CF)+R.farm(ACFT,B) | age+farm(A,B,CT,F) | age+soc(Ad) | ss | 2115.1 | 2207.5 | 43 | 124.6 |
| season | (K&J,Ad).farm(A,BCF,T) | age | farm | K&J+socS(Ad)+socU(Ad).farm(AT,B,CF)+R.farm(ACFT,B) | age+farm(AF,B,CT) | age+soc(Ad) | ss | 2115.3 | 2205.3 | 42 | 122.4 |
| season | (K&J,Ad).farm(A,BCF,T) | age | farm | K&J+socS(Ad)+socU(Ad).farm(AT,B,CF)+R.farm(ACFT,B) | [farm(AF,B)+age] +farm(CT) | age+soc(Ad) | ss | 2113.8 | 2203.8 | 42 | 120.9 |
| season | (K&J,Ad).farm(A,BCF,T) | age | farm | K&J+socS(Ad)+socU(Ad).farm(AT,B,CF)+R.farm(ACFT,B) | [farm(AF,B)+(K&J)]+farm(CT)&Ad | age+soc(Ad) | ss | 2113.8 | 2201.6 | 41 | 118.6 |
| season | (K&J,Ad).farm(A,BCF,T) | age | farm | K&J+socS(Ad)+socU(Ad).farm(AT,B,CF)+R.farm(ACFT,B) | [farm(AF,B)+(K&J)]+farm(CT)&Ad | (K,J&U)+soc(Ad) | ss | 2093.8 | 2179.3 | 40 | 96.3 |
| season | (K&J,Ad).farm(A,BCF,T) | age | farm | K&J+socS(Ad)+socU(Ad).farm(AT,B,CF)+R.farm(ACFT,B) | [farm(AF,B)+(K&J)]+farm(CT)&Ad | (K,J&U,Ad)+soc | ss | 2092.0 | 2177.5 | 40 | 94.5 |
| season | (K&J,Ad).farm(A,BCF,T) | age | farm | K&J+socS(Ad)+socU(Ad).farm(AT,B,CF)+R.farm(ACFT,B) | [farm(AF,B)+(K&J)]+farm(CT)&Ad | K+[(J&U,Ad)+soc] | ss | 2092.7 | 2180.4 | 41 | 97.5 |
| season | (K&J,Ad).farm(A,BCF,T) | age | farm | K&J+socS(Ad)+socU(Ad).farm(AT,B,CF)+R.farm(ACFT,B) | [farm(AF,B)+(K&J)]+farm(CT)&Ad | (K,J&U,Ad)+soc.season | ss | 2045.5 | 2156.5 | 51 | 73.6 |
| season | (K&J,Ad).farm(A,BCF,T) | age | farm | K&J+socS(Ad)+socU(Ad).farm(AT,B,CF)+R.farm(ACFT,B) | [farm(AF,B)+(K&J)]+farm(CT)&Ad | (K,J&U,Ad)+soc+season | ss | 2080.6 | 2172.9 | 43 | 90.0 |
| season | (K&J,Ad).farm(A,BCF,T) | age | farm | K&J+socS(Ad)+socU(Ad).farm(AT,B,CF)+R.farm(ACFT,B) | [farm(AF,B)+(K&J)]+farm(CT)&Ad | (K,J&U,Ad)+soc.gender | ss | 2087.7 | 2182.3 | 44 | 99.4 |
| season | (K&J,Ad).farm(A,BCF,T) | age | farm | K&J+socS(Ad)+socU(Ad).farm(AT,B,CF)+R.farm(ACFT,B) | [farm(AF,B)+(K&J)]+farm(CT)&Ad | (K,J&U,Ad)+soc+gender | ss | 2091.4 | 2179.1 | 41 | 96.2 |
| season | (K&J,Ad).farm(A,BCF,T) | age | farm | K&J+socS(Ad)+socU(Ad).farm(AT,B,CF)+R.farm(ACFT,B) | [farm(AF,B)+(K&J)]+farm(CT)&Ad | (K,J&U,Ad)+soc.farm | ss | 2045.4 | 2168.3 | 56 | 85.4 |
| season | (K&J,Ad).farm(A,BCF,T) | age | farm | K&J+socS(Ad)+socU(Ad).farm(AT,B,CF)+R.farm(ACFT,B) | [farm(AF,B)+(K&J)]+farm(CT)&Ad | (K,J&U,Ad)+soc+farm | ss | 2074.0 | 2168.6 | 44 | 85.7 |
| season | (K&J,Ad).farm(A,BCF,T) | age | farm | K&J+socS(Ad)+socU(Ad).farm(AT,B,CF)+R.farm(ACFT,B) | [farm(AF,B)+(K&J)]+farm(CT)&Ad | [[(K).soc+(J&U,Ad).socB].season]+[(J&U,Ad).socS] | ss | 2058.6 | 2172.0 | 52 | 89.1 |
| season | (K&J,Ad).farm(A,BCF,T) | age | farm | K&J+socS(Ad)+socU(Ad).farm(AT,B,CF)+R.farm(ACFT,B) | [farm(AF,B)+(K&J)]+farm(CT)&Ad | [(K).season(Sp,SuW,Au)+(J&U).season(SpAu,Su,W)+(Ad).season(Sp,SuAu,W)]+soc | ss | 2050.0 | 2149.3 | 46 | 66.3 |
| season | (K&J,Ad).farm(A,BCF,T) | age | farm | K&J+socS(Ad)+socU(Ad).farm(AT,B,CF)+R.farm(ACFT,B) | [farm(AF,B)+(K&J)]+farm(CT)&Ad | [(K).season(Sp,SuW,Au)+(J&U).season(SpAu,Su,W)+(Ad).season(Sp&W,Su&A)]+soc | ss | 2050.2 | 2147.1 | 45 | 64.2 |
| season | (K&J,Ad).farm(A,BCF,T) | age | farm | K&J+socS(Ad)+socU(Ad).farm(AT,B,CF)+R.farm(ACFT,B) | [farm(AF,B)+(K&J)]+farm(CT)&Ad | [[(K).season(Sp,SuW,Au)+(J&U).season(SpAu,Su,W)+(Ad).season(SpW,SuAu)]+soc]+farm(A,F,C,B&T) | ss | 2028.3 | 2132.2 | 48 | 49.3 |
| season | (K&J,Ad).farm(A,BCF,T) | age | farm | K&J+socS(Ad)+socU(Ad).farm(AT,B,CF)+R.farm(ACFT,B) | [farm(AF,B)+(K&J)]+farm(CT)&Ad | [[(K).season(Sp,SuW,Au)+(J&U).season(SpAu,Su,W)+(Ad).season(SpW,SuAu)]+soc]+farm(ABT,F,C) | ss | 2028.4 | 2130.0 | 47 | 47.1 |
| season | (K&J,Ad).farm(A,BCF,T) | age | farm | K&J+socS(Ad)+socU(Ad).farm(AT,B,CF)+R.farm(ACFT,B) | [farm(AF,B)+(K&J)]+farm(CT)&Ad | [[(K).season(Sp,Su&W,A)+(J&U).season(Sp A,Su,W)+(A).season(Sp&W,Su&A)]+soc]+gender | ss | 2049.7 | 2149.0 | 46 | 66.0 |
| season | (K&J,Ad).farm(A,BCF,T) | age | farm | K&J+socS(Ad)+socU(Ad).farm(AT,B,CF)+R.farm(ACFT,B) | [farm(AF,B)+(K&J)]+farm(CT)&Ad | [[(K).season(Sp,SuW,Au)+(J&U).season(SpAu,Su,W)+(Ad).season(SpW,SuAu)]+soc]+farm(ABT,F,C) | ss.t(1 2 3, 4 5 6 7 8) | 2007.9 | 2118.9 | 51 | 36.0 |
| season | (K&J,Ad).farm(A,BCF,T) | age | farm | K&J+socS(Ad)+socU(Ad).farm(AT,B,CF)+R.farm(ACFT,B) | [farm(AF,B)+(K&J)]+farm(CT)&Ad | [[(K).season(Sp,SuW,Au)+(J&U).season(SpAu,Su,W)+(Ad).season(SpW,SuAu)]+soc]+farm(ABT,F,C) | ss.farm(ABF,CT) | 2008.6 | 2119.6 | 51 | 36.7 |
| season | (K&J,Ad).farm(A,BCF,T) | age | farm | K&J+socS(Ad)+socU(Ad).farm(AT,B,CF)+R.farm(ACFT,B) | [farm(AF,B)+(K&J)]+farm(CT)&Ad | [[(K).season(Sp,SuW,Au)+(J&U).season(SpAu,Su,W)+(Ad).season(SpW,SuAu)]+soc]+farm(ABT,F,C) | ssN+[ssP.farm(ABF,CT)] | 2008.9 | 2115.2 | 49 | 32.3 |
| season | (K&J,Ad).farm(A,BCF,T) | age | farm | K&J+socS(Ad)+socU(Ad).farm(AT,B,CF)+R.farm(ACFT,B) | [farm(AF,B)+(K&J)]+farm(CT)&Ad | [[(K).season(Sp,SuW,Au)+(J&U).season(SpAu,Su,W)+(Ad).season(SpW,SuAu)]+soc]+farm(ABT,F,C) | [ssN+[ssP.farm(ABF,CT)]]+t(1 2 3, 4 5 6 7 8) | 1993.1 | 2101.8 | 50 | 18.8 |
| season | (K&J,Ad).farm(A,BCF,T) | age.season | farm | K&J+socS(Ad)+socU(Ad).farm(AT,B,CF)+R.farm(ACFT,B) | [farm(AF,B)+(K&J)]+farm(CT)&Ad | [[(K).season(Sp,SuW,Au)+(J&U).season(SpAu,Su,W)+(Ad).season(SpW,SuAu)]+soc]+farm(ABT,F,C) | [ssN+[ssP.farm(ABF,CT)]]+t(1 2 3, 4 5 6 7 8) | 1973.6 | 2001.4 | 58 | -81.6 |
| season | (K&J,Ad).farm(A,BCF,T) | age+season | farm | K&J+socS(Ad)+socU(Ad).farm(AT,B,CF)+R.farm(ACFT,B) | [farm(AF,B)+(K&J)]+farm(CT)&Ad | [[(K).season(Sp,SuW,Au)+(J&U).season(SpAu,Su,W)+(Ad).season(SpW,SuAu)]+soc]+farm(ABT,F,C) | [ssN+[ssP.farm(ABF,CT)]]+t(1 2 3, 4 5 6 7 8) | 1984.7 | 2000.4 | 53 | -82.5 |
| season | (K&J,Ad).farm(A,BCF,T) | age.gender | farm | K&J+socS(Ad)+socU(Ad).farm(AT,B,CF)+R.farm(ACFT,B) | [farm(AF,B)+(K&J)]+farm(CT)&Ad | [[(K).season(Sp,SuW,Au)+(J&U).season(SpAu,Su,W)+(Ad).season(SpW,SuAu)]+soc]+farm(ABT,F,C) | [ssN+[ssP.farm(ABF,CT)]]+t(1 2 3, 4 5 6 7 8) | 1987.5 | 2103.3 | 53 | 20.4 |
| season | (K&J,Ad).farm(A,BCF,T) | age+gender | farm | K&J+socS(Ad)+socU(Ad).farm(AT,B,CF)+R.farm(ACFT,B) | [farm(AF,B)+(K&J)]+farm(CT)&Ad | [[(K).season(Sp,SuW,Au)+(J&U).season(SpAu,Su,W)+(Ad).season(SpW,SuAu)]+soc]+farm(ABT,F,C) | [ssN+[ssP.farm(ABF,CT)]]+t(1 2 3, 4 5 6 7 8) | 1992.4 | 2103.4 | 51 | 20.5 |
| season | (K&J,Ad).farm(A,BCF,T) | age+farm | farm | K&J+socS(Ad)+socU(Ad).farm(AT,B,CF)+R.farm(ACFT,B) | [farm(AF,B)+(K&J)]+farm(CT)&Ad | [[(K).season(Sp,SuW,Au)+(J&U).season(SpAu,Su,W)+(Ad).season(SpW,SuAu)]+soc]+farm(ABT,F,C) | [ssN+[ssP.farm(ABF,CT)]]+t(1 2 3, 4 5 6 7 8) | 1988.9 | 2107.0 | 54 | 24.1 |
| season | (K&J,Ad).farm(A,BCF,T) | age+season(Sp,Su,AuW) | farm | K&J+socS(Ad)+socU(Ad).farm(AT,B,CF)+R.farm(ACFT,B) | [farm(AF,B)+(K&J)]+farm(CT)&Ad | [[(K).season(Sp,SuW,Au)+(J&U).season(SpAu,Su,W)+(Ad).season(SpW,SuAu)]+soc]+farm(ABT,F,C) | [ssN+[ssP.farm(ABF,CT)]]+t(1 2 3, 4 5 6 7 8) | 1984.8 | 2098.2 | 52 | 15.3 |
| season | (K&J,Ad).farm(A,BCF,T) | K.season(SpAu,Su,W)+J.season(SpSu,AuW)+Ad | farm | K&J+socS(Ad)+socU(Ad).farm(AT,B,CF)+R.farm(ACFT,B) | [farm(AF,B)+(K&J)]+farm(CT)&Ad | [[(K).season(Sp,SuW,Au)+(J&U).season(SpAu,Su,W)+(Ad).season(SpW,SuAu)]+soc]+farm(ABT,F,C) | [ssN+[ssP.farm(ABF,CT)]]+t(1 2 3, 4 5 6 7 8) | 1982.4 | 2098.1 | 53 | 15.2 |
| season | (K&J,Ad).farm(A,BCF,T) | K.season(SpAu,Su,W)+(J,Ad) | farm | K&J+socS(Ad)+socU(Ad).farm(AT,B,CF)+R.farm(ACFT,B) | [farm(AF,B)+(K&J)]+farm(CT)&Ad | [[(K).season(Sp,SuW,Au)+(J&U).season(SpAu,Su,W)+(Ad).season(SpW,SuAu)]+soc]+farm(ABT,F,C) | [ssN+[ssP.farm(ABF,CT)]]+t(1 2 3, 4 5 6 7 8) | 1987.2 | 2100.6 | 52 | 17.7 |
| season | (K&J,Ad).farm(A,BCF,T) | age+season(Sp,Su,AuW) | age.farm | K&J+socS(Ad)+socU(Ad).farm(AT,B,CF)+R.farm(ACFT,B) | [farm(AF,B)+(K&J)]+farm(CT)&Ad | [[(K).season(Sp,SuW,Au)+(J&U).season(SpAu,Su,W)+(Ad).season(SpW,SuAu)]+soc]+farm(ABT,F,C) | [ssN+[ssP.farm(ABF,CT)]]+t(1 2 3, 4 5 6 7 8) | 1975.7 | 2110.8 | 61 | 27.9 |
| season | (K&J,Ad).farm(A,BCF,T) | age+season(Sp,Su,AuW) | age(KJ,Ad).farm | K&J+socS(Ad)+socU(Ad).farm(AT,B,CF)+R.farm(ACFT,B) | [farm(AF,B)+(K&J)]+farm(CT)&Ad | [[(K).season(Sp,SuW,Au)+(J&U).season(SpAu,Su,W)+(Ad).season(SpW,SuAu)]+soc]+farm(ABT,F,C) | [ssN+[ssP.farm(ABF,CT)]]+t(1 2 3, 4 5 6 7 8) | 1976.0 | 2101.4 | 57 | 18.5 |
| season | (K&J,Ad).farm(A,BCF,T) | age+season(Sp,Su,AuW) | farm+season | K&J+socS(Ad)+socU(Ad).farm(AT,B,CF)+R.farm(ACFT,B) | [farm(AF,B)+(K&J)]+farm(CT)&Ad | [[(K).season(Sp,SuW,Au)+(J&U).season(SpAu,Su,W)+(Ad).season(SpW,SuAu)]+soc]+farm(ABT,F,C) | [ssN+[ssP.farm(ABF,CT)]]+t(1 2 3, 4 5 6 7 8) | 1978.4 | 2098.9 | 55 | 16.0 |
| season | (K&J,Ad).farm(A,BCF,T) | age+season(Sp,Su,AuW) | farm+season(Sp,Su,AuW) | K&J+socS(Ad)+socU(Ad).farm(AT,B,CF)+R.farm(ACFT,B) | [farm(AF,B)+(K&J)]+farm(CT)&Ad | [[(K).season(Sp,SuW,Au)+(J&U).season(SpAu,Su,W)+(Ad).season(SpW,SuAu)]+soc]+farm(ABT,F,C) | [ssN+[ssP.farm(ABF,CT)]]+t(1 2 3, 4 5 6 7 8) | 1978.4 | 2096.5 | 54 | 13.6 |
| season | (K&J,Ad).farm(A,BCF,T) | age+season(Sp,Su,AuW) | farm+season(SpSu,AuW) | K&J+socS(Ad)+socU(Ad).farm(AT,B,CF)+R.farm(ACFT,B) | [farm(AF,B)+(K&J)]+farm(CT)&Ad | [[(K).season(Sp,SuW,Au)+(J&U).season(SpAu,Su,W)+(Ad).season(SpW,SuAu)]+soc]+farm(ABT,F,C) | [ssN+[ssP.farm(ABF,CT)]]+t(1 2 3, 4 5 6 7 8) | 1978.9 | 2095.0 | 53 | 12.0 |
| season | (K&J,Ad).farm(A,BCF,T) | age+season(Sp,Su,AuW) | farm+gender | K&J+socS(Ad)+socU(Ad).farm(AT,B,CF)+R.farm(ACFT,B) | [farm(AF,B)+(K&J)]+farm(CT)&Ad | [[(K).season(Sp,SuW,Au)+(J&U).season(SpAu,Su,W)+(Ad).season(SpW,SuAu)]+soc]+farm(ABT,F,C) | [ssN+[ssP.farm(ABF,CT)]]+t(1 2 3, 4 5 6 7 8) | 1884.2 | 2099.9 | 53 | 17.0 |
| season | (K&J,Ad).farm(A,BCF,T) | age+season(Sp,Su,AuW) | [age(KJ,Ad).farm]+season(SpSu,AuW) | K&J+socS(Ad)+socU(Ad).farm(AT,B,CF)+R.farm(ACFT,B) | [farm(AF,B)+(K&J)]+farm(CT)&Ad | [[(K).season(Sp,SuW,Au)+(J&U).season(SpAu,Su,W)+(Ad).season(SpW,SuAu)]+soc]+farm(ABT,F,C) | [ssN+[ssP.farm(ABF,CT)]]+t(1 2 3, 4 5 6 7 8) | 1966.6 | 2094.3 | 58 | 11.4 |
| season | (K&J,Ad).farm(A,BCF,T) | age+season(Sp,Su,AuW) | [farm(A)+age(KJ,Ad).farm(B,C,F,T)]+season(SpSu,AuW) | K&J+socS(Ad)+socU(Ad).farm(AT,B,CF)+R.farm(ACFT,B) | [farm(AF,B)+(K&J)]+farm(CT)&Ad | [[(K).season(Sp,SuW,Au)+(J&U).season(SpAu,Su,W)+(Ad).season(SpW,SuAu)]+soc]+farm(ABT,F,C) | [ssN+[ssP.farm(ABF,CT)]]+t(1 2 3, 4 5 6 7 8) | 1967.2 | 2092.6 | 57 | 9.6 |
| season | (K&J,Ad).farm(A,BCF,T) | age+season(Sp,Su,AuW) | [farm(AF)+age(KJ,Ad).farm(B,C,T)]+season(SpSu,AuW) | K&J+socS(Ad)+socU(Ad).farm(AT,B,CF)+R.farm(ACFT,B) | [farm(AF,B)+(K&J)]+farm(CT)&Ad | [[(K).season(Sp,SuW,Au)+(J&U).season(SpAu,Su,W)+(Ad).season(SpW,SuAu)]+soc]+farm(ABT,F,C) | [ssN+[ssP.farm(ABF,CT)]]+t(1 2 3, 4 5 6 7 8) | 1968.9 | 2091.9 | 56 | 8.9 |
| season | (K&J,Ad).farm(A,BCF,T) | age+season(Sp,Su,AuW) | [farm(AF)+age(KJ,Ad).farm(BT,C)]+season(SpSu,AuW) | K&J+socS(Ad)+socU(Ad).farm(AT,B,CF)+R.farm(ACFT,B) | [farm(AF,B)+(K&J)]+farm(CT)&Ad | [[(K).season(Sp,SuW,Au)+(J&U).season(SpAu,Su,W)+(Ad).season(SpW,SuAu)]+soc]+farm(ABT,F,C) | [ssN+[ssP.farm(ABF,CT)]]+t(1 2 3, 4 5 6 7 8) | 1969.2 | 2085.0 | 53 | 2.1 |
| season | (K&J,Ad).farm(A,BCF,T) | age+season(Sp,Su,AuW) | farm(AF).season(Sp,Su,AuW)+[age(KJ,A).farm(BT,C)].season(SpSu,AuW)] | K&J+socS(Ad)+socU(Ad).farm(AT,B,CF)+R.farm(ACFT,B) | [farm(AF).(K&J)]+[farm(B).(K,J)]+[farm(CT)&farm(AFB).A] | [[(K).season(Sp,SuW,Au)+(J&U).season(SpAu,Su,W)+(Ad).season(SpW,SuAu)]+soc]+farm(ABT,F,C) | [ssN+[ssP.farm(ABF,CT)]]+t(1 2 3, 4 5 6 7 8) | 1963.9 | 2091.7 | 58 | 8.8 |
| season | (K&J,Ad).farm(A,BCF,T) | age+season(Sp,Su,AuW) | [farm(AF)+age(KJ,A).farm(BT,C)]+season(SpSu,AuW)+gender] | K&J+socS(Ad)+socU(Ad).farm(AT,B,CF)+R.farm(ACFT,B) | [farm(AF).(K&J)]+[farm(B).(K,J)]+[farm(CT)&farm(AFB).A] | [[(K).season(Sp,SuW,Au)+(J&U).season(SpAu,Su,W)+(Ad).season(SpW,SuAu)]+soc]+farm(ABT,F,C) | [ssN+[ssP.farm(ABF,CT)]]+t(1 2 3, 4 5 6 7 8) | 1968.6 | 2086.7 | 54 | 3.8 |
| season | (K&J,Ad).farm(A,BCF,T) | age+season(Sp,Su,AuW) | farm(AF)+age(KJ,A).farm(BT,C) | K&J+socS(Ad)+socU(Ad).farm(AT,B,CF)+R.farm(ACFT,B) | [farm(AF).(K&J)]+[farm(B).(K,J)]+[farm(CT)&farm(AFB).A] | [[(K).season(Sp,SuW,Au)+(J&U).season(SpAu,Su,W)+(Ad).season(SpW,SuAu)]+soc]+farm(ABT,F,C) | [ssN+[ssP.farm(ABF,CT)]]+t(1 2 3, 4 5 6 7 8) | 1919.0 | 2092.3 | 52 | 9.4 |
| season | farm(A,BCF, T) | age+season(Sp,Su,AuW) | [farm(AF)+age(KJ,A).farm(BT,C)]+season(SpSu,AuW) | K&J+socS(Ad)+socU(Ad).farm(AT,B,CF)+R.farm(ACFT,B) | [farm(AF).(K&J)]+[farm(B).(K,J)]+[farm(CT)&farm(AFB).A] | [[(K).season(Sp,SuW,Au)+(J&U).season(SpAu,Su,W)+(Ad).season(SpW,SuAu)]+soc]+farm(ABT,F,C) | [ssN+[ssP.farm(ABF,CT)]]+t(1 2 3, 4 5 6 7 8) | 2013.4 | 2122.0 | 50 | 39.1 |
| season | (K&J,Ad).farm(A,BCF,T) | age+season(SpSu, AuW) | [farm(AF)+age(KJ,A).farm(BT,C)]+season(SpSu,AuW) | K&J+socS(Ad)+socU(Ad).farm(AT,B,CF)+R.farm(ACFT,B) | [farm(AF).(K&J)]+[farm(B).(K,J)]+[farm(CT)&farm(AFB).A] | [[(K).season(Sp,SuW,Au)+(J&U).season(SpAu,Su,W)+(Ad).season(SpW,SuAu)]+soc]+farm(ABT,F,C) | [ssN+[ssP.farm(ABF,CT)]]+t(1 2 3, 4 5 6 7 8) | 1977.4 | 2090.8 | 52 | 7.8 |
| season | (K&J,Ad).farm(A,BCF,T) | age+season(Sp,SuAuW) | [farm(AF)+age(KJ,A).farm(BT,C)]+season(SpSu,AuW) | K&J+socS(Ad)+socU(Ad).farm(AT,B,CF)+R.farm(ACFT,B) | [farm(AF).(K&J)]+[farm(B).(K,J)]+[farm(CT)&farm(AFB).A] | [[(K).season(Sp,SuW,Au)+(J&U).season(SpAu,Su,W)+(Ad).season(SpW,SuAu)]+soc]+farm(ABT,F,C) | [ssN+[ssP.farm(ABF,CT)]]+t(1 2 3, 4 5 6 7 8) | 1971.0 | 2084.4 | 51 | 1.5 |
| season | (K&J,Ad).farm(A,BCF,T) | age | [farm(AF)+age(KJ,A).farm(BT,C)]+season(SpSu,AuW) | K&J+socS(Ad)+socU(Ad).farm(AT,B,CF)+R.farm(ACFT,B) | [farm(AF).(K&J)]+[farm(B).(K,J)]+[farm(CT)&farm(AFB).A] | [[(K).season(Sp,SuW,Au)+(J&U).season(SpAu,Su,W)+(Ad).season(SpW,SuAu)]+soc]+farm(ABT,F,C) | [ssN+[ssP.farm(ABF,CT)]]+t(1 2 3, 4 5 6 7 8) | 1977.5 | 2088.5 | 51 | 5.6 |
| season | (K&J,Ad).farm(A,BCF,T) | age+season(Sp,Su,AuW) | [farm(AF)+age(KJ,A).farm(BT,C)]+season(SpSu,AuW) | K&J+socS(ad)+socU(ad).farm(AT,B,CF)+R | [farm(AF).(K&J)]+[farm(B).(K,J)]+[farm(CT)&farm(AFB).A] | [[(K).season(Sp,SuW,Au)+(J&U).season(SpAu,Su,W)+(Ad).season(SpW,SuAu)]+soc]+farm(ABT,F,C) | [ssN+[ssP.farm(ABF,CT)]]+t(1 2 3, 4 5 6 7 8) | 1996.8 | 2110.2 | 52 | 27.2 |
| season | (K&J,Ad).farm(A,BCF,T) | age+season(Sp,Su,AuW) | [farm(AF)+age(KJ,A).farm(BT,C)]+season(SpSu,AuW) | K&J+socS(ad)+socU(ad)+R.farm(ACFT,B) | [farm(AF).(K&J)]+[farm(B).(K,J)]+[farm(CT)&farm(AFB).A] | [[(K).season(Sp,SuW,Au)+(J&U).season(SpAu,Su,W)+(Ad).season(SpW,SuAu)]+soc]+farm(ABT,F,C) | [ssN+[ssP.farm(ABF,CT)]]+t(1 2 3, 4 5 6 7 8) | 1980.0 | 2091.3 | 51 | 8.4 |
| season | (K&J,Ad).farm(A,BCF,T) | age+season(Sp,Su,AuW) | [farm(AF)+age(KJ,A).farm(BT,C)]+season(SpSu,AuW) | K&J+socS(Ad)+socU(Ad).farm(AT,B,CF)+R.farm(ACFT,B) | [farm(AF,B).(K&J)]+[farm(CT)&farm(AFB).A] | [[(K).season(Sp,SuW,Au)+(J&U).season(SpAu,Su,W)+(Ad).season(SpW,SuAu)]+soc]+farm(ABT,F,C) | [ssN+[ssP.farm(ABF,CT)]]+t(1 2 3, 4 5 6 7 8) | 2048.6 | 2161.9 | 51 | 79.0 |
| season | (K&J,Ad).farm(A,BCF,T) | age+season(Sp,Su,AuW) | [farm(AF)+age(KJ,A).farm(BT,C)]+season(SpSu,AuW) | K&J+socS(Ad)+socU(Ad).farm(AT,B,CF)+R.farm(ACFT,B) | [farm(B).(K&J)]+[farm(CTAF)&farm(B).A] | [[(K).season(Sp,SuW,Au)+(J&U).season(SpAu,Su,W)+(Ad).season(SpW,SuAu)]+soc]+farm(ABT,F,C) | [ssN+[ssP.farm(ABF,CT)]]+t(1 2 3, 4 5 6 7 8) | 1991.3 | 2104.6 | 52 | 21.7 |
| season | (K&J,Ad).farm(A,BCF,T) | age+season(Sp,Su,AuW) | [farm(AF)+age(KJ,A).farm(BT,C)]+season(SpSu,AuW) | K&J+socS(Ad)+socU(Ad).farm(AT,B,CF)+R.farm(ACFT,B) | [farm(AF).(K&J)]+[farm(B).(K,J)]+[farm(CT)&farm(AFB).A] | [[(K).season(Sp,SuW,Au)+(J&U).season(SpAu,Su,W)+(Ad).season(SpW,SuAu)]+soc] | [ssN+[ssP.farm(ABF,CT)]]+t(1 2 3, 4 5 6 7 8) | 1991.0 | 2102.0 | 51 | 19.1 |
| season | (K&J,Ad).farm(A,BCF,T) | age+season(Sp,Su,AuW) | [farm(AF)+age(KJ,A).farm(BT,C)]+season(SpSu,AuW) | K&J+socS(Ad)+socU(Ad).farm(AT,B,CF)+R.farm(ACFT,B) | [farm(AF).(K&J)]+[farm(B).(K,J)]+[farm(CT)&farm(AFB).A] | [[(K).season(Sp,SuW,Au)+(J&U).season(SpAu,Su,W)+(Ad)]+soc]+farm(ABT,F,C) | [ssN+[ssP.farm(ABF,CT)]]+t(1 2 3, 4 5 6 7 8) | 1990.7 | 2104.0 | 52 | 21.1 |
| season | (K&J,Ad).farm(A,BCF,T) | age+season(Sp,Su,AuW) | [farm(AF)+age(KJ,A).farm(BT,C)]+season(SpSu,AuW) | K&J+socS(Ad)+socU(Ad).farm(AT,B,CF)+R.farm(ACFT,B) | [farm(AF).(K&J)]+[farm(B).(K,J)]+[farm(CT)&farm(AFB).A] | [[(K).season(Sp,SuW,Au)+(J&U).season(SpAu, SuW)+(Ad).season(SpW,SuAu)]+soc]+farm(ABT,F,C) | [ssN+[ssP.farm(ABF,CT)]]+t(1 2 3, 4 5 6 7 8) | 1970.1 | 2083.5 | 52 | 0.6 |
| season | (K&J,Ad).farm(A,BCF,T) | age+season(Sp,Su,AuW) | [farm(AF)+age(KJ,A).farm(BT,C)]+season(SpSu,AuW) | K&J+socS(Ad)+socU(Ad).farm(AT,B,CF)+R.farm(ACFT,B) | [farm(AF).(K&J)]+[farm(B).(K,J)]+[farm(CT)&farm(AFB).A] | [[(K).season(Sp,SuW,Au)+(J&U)+(Ad).season(SpW,SuAu)]+soc]+farm(ABT,F,C) | [ssN+[ssP.farm(ABF,CT)]]+t(1 2 3, 4 5 6 7 8) | 1974.4 | 2085.4 | 51 | 2.4 |
| season | (K&J,Ad).farm(A,BCF,T) | age+season(Sp,Su,AuW) | [farm(AF)+age(KJ,A).farm(BT,C)]+season(SpSu,AuW) | K&J+socS(Ad)+socU(Ad).farm(AT,B,CF)+R.farm(ACFT,B) | [farm(AF).(K&J)]+[farm(B).(K,J)]+[farm(CT)&farm(AFB).A] | [[(K).season(SpAu,SuW)+(J&U).season(SpAu,SuW)+(Ad).season(SpW,SuAu)]+soc]+farm(ABT,F,C) | [ssN+[ssP.farm(ABF,CT)]]+t(1 2 3, 4 5 6 7 8) | 1975.4 | 2086.4 | 51 | 3.5 |
| season | (K&J,Ad).farm(A,BCF,T) | age+season(Sp,Su,AuW) | [farm(AF)+age(KJ,A).farm(BT,C)]+season(SpSu,AuW) | K&J+socS(Ad)+socU(Ad).farm(AT,B,CF)+R.farm(ACFT,B) | [farm(AF).(K&J)]+[farm(B).(K,J)]+[farm(CT)&farm(AFB).A] | [[(K).season(SpAu,SuW)+(J&U).season(SpAu,SuW)+(Ad).season(SpW,SuAu)]+soc]+farm(ABT,FC) | [ssN+[ssP.farm(ABF,CT)]]+t(1 2 3, 4 5 6 7 8) | 1988.2 | 2099.2 | 51 | 16.3 |
| season | (K&J,Ad).farm(A,BCF,T) | age+season(Sp,Su,AuW) | [farm(AF)+age(KJ,A).farm(BT,C)]+season(SpSu,AuW) | K&J+socS(Ad)+socU(Ad).farm(AT,B,CF)+R.farm(ACFT,B) | [farm(AF).(K&J)]+[farm(B).(K,J)]+[farm(CT)&farm(AFB).A] | [[(K).season(Sp,SuW,Au)+(J&U).season(SpAu,SuW)+(Ad).season(SpW,SuAu)]+soc]+farm(ABT,F,C) | [ssN+[ssP.farm(ABF,CT)]] | 1984.1 | 2095.1 | 51 | 12.2 |
| season | (K&J,Ad).farm(A,BCF,T) | age+season(Sp,Su,AuW) | [farm(AF)+age(KJ,A).farm(BT,C)]+season(SpSu,AuW) | K&J+socS(Ad)+socU(Ad).farm(AT,B,CF)+R.farm(ACFT,B) | [farm(AF).(K&J)]+[farm(B).(K,J)]+[farm(CT)&farm(AFB).A] | [[(K).season(Sp,SuW,Au)+(J&U).season(SpAu,SuW)+(Ad).season(SpW,SuAu)]+soc]+farm(ABT,F,C) | ss+t(1 2 3, 4 5 6 7 8) | 1991.3 | 2100.0 | 50 | 17.0 |
| **season** | **(K&J,Ad).farm(A,BCF,T)** | **age+season(Sp,SuAuW)** | **[farm(AF)+age(KJ,A).farm(BT,C)]+season(SpSu,AuW)** | **K&J+socS(Ad)+socU(Ad).farm(AT,B,CF)+R.farm(ACFT,B)** | **[farm(AF).(K&J)]+[farm(B).(K,J)]+[farm(CT)&farm(AFB).A]** | **[[(K).season(Sp,SuW,Au)+(J&U).season(SpAu,SuW)+(Ad).season(SpW,SuAu)]+soc]+farm(ABT,F,C)** | **[ssN+[ssP.farm(ABF,CT)]]+t(1 2 3, 4 5 6 7 8)** | **1971.9** | **2082.9** | **51** | **0.0** |

**Table S2.** Estimated parameters from the selected model. *Abbreviations*: LCL, lower confidence limit; UCL, upper confidence limit; SE, standard error.

| **Parameters** | **Effects** | **Estimate** | **LCL** | **UCL** | **SE** |
| --- | --- | --- | --- | --- | --- |
| Initial age (*Ia*) | Kit - Spring | 0.23 | NA | NA | NA |
| Initial age (*Ia*) | Juv - Spring | 0.00 | 0.00 | 0.00 | 0.00 |
| Initial age (*Ia*) | Ad - Spring | 0.77 | 0.66 | 0.86 | 0.05 |
| Initial age (*Ia*) | Kit - Summer | 0.85 | NA | NA | NA |
| Initial age (*Ia*) | Juv - Summer | 0.11 | 0.04 | 0.29 | 0.06 |
| Initial age (*Ia*) | Ad - Summer | 0.04 | 0.01 | 0.22 | 0.04 |
| Initial age (*Ia*) | Kit - Autumn | 0.53 | NA | NA | NA |
| Initial age (*Ia*) | Juv - Autumn | 0.25 | 0.14 | 0.39 | 0.06 |
| Initial age (*Ia*) | Ad - Autumn | 0.23 | 0.13 | 0.37 | 0.06 |
| Initial age (*Ia*) | Kit - Winter | 0.15 | NA | NA | NA |
| Initial age (*Ia*) | Juv - Winter | 0.60 | 0.31 | 0.83 | 0.15 |
| Initial age (*Ia*) | Ad - Winter | 0.25 | 0.08 | 0.55 | 0.13 |
| Initial serologic status (*Iss*) | Kit & Juv - Positive - Farm A | 0.35 | 0.18 | 0.56 | 0.10 |
| Initial serologic status (*Iss*) | Ad - Positive - Farm A | 0.85 | 0.32 | 0.98 | 0.16 |
| Initial serologic status (*Iss*) | Kit & Juv - Positive - Farm B, C & F | 0.03 | 0.00 | 0.25 | 0.04 |
| Initial serologic status (*Iss*) | Ad - Positive - Farm B, C & F | 0.83 | 0.62 | 0.94 | 0.08 |
| Initial serologic status (*Iss*) | Kit & Juv - Positive - Farm T | 0.00 | 0.00 | 0.00 | 0.00 |
| Initial serologic status (*Iss*) | Ad - Positive - Farm T | 0.00 | 0.00 | 0.00 | 0.00 |
| Survival (*S*) | Kit - Spring | 0.44 | 0.29 | 0.61 | 0.08 |
| Survival (*S*) | Juv - Spring | 0.71 | 0.50 | 0.86 | 0.10 |
| Survival (*S*) | Ad - Spring | 0.84 | 0.77 | 0.89 | 0.03 |
| Survival (*S*) | Kit - Summer& Autumn & Winter | 0.63 | 0.51 | 0.74 | 0.07 |
| Survival (*S*) | Juv - Summer& Autumn & Winter | 0.84 | 0.72 | 0.92 | 0.05 |
| Survival (*S*) | Ad - Summer& Autumn & Winter | 0.92 | 0.88 | 0.95 | 0.02 |
| Seroconversion (*Ψ*) | All - Farm A & F - Spring & Summer | 0.27 | 0.12 | 0.49 | 0.10 |
| Seroconversion (*Ψ*) | All - Farm A & F - Autumn & Winter | 0.73 | 0.50 | 0.88 | 0.10 |
| Seroconversion (*Ψ*) | Kit & Juv - Farm B &T- Spring & Summer | 0.00 | 0.00 | 0.00 | 0.00 |
| Seroconversion (*Ψ*) | Ad - Farm B &T - Spring & Summer | 0.04 | 0.01 | 0.13 | 0.02 |
| Seroconversion (*Ψ*) | Kit & Juv - Farm B &T - Autumn & Winter | 0.00 | 0.00 | 0.00 | 0.00 |
| Seroconversion (*Ψ*) | Ad - Farm B &T - Autumn & Winter | 0.21 | 0.09 | 0.41 | 0.08 |
| Seroconversion (*Ψ*) | Kit & Juv - Farm C - Spring & Summer | 0.17 | 0.05 | 0.45 | 0.10 |
| Seroconversion (*Ψ*) | Ad - Farm C - Spring & Summer | 0.00 | 0.00 | 0.00 | 0.00 |
| Seroconversion (*Ψ*) | Kit & Juv - Farm C - Autumn & Winter | 0.59 | 0.21 | 0.89 | 0.21 |
| Seroconversion (*Ψ*) | Ad - Farm C - Autumn & Winter | 0.00 | 0.00 | 0.00 | 0.00 |
| Detection (*β*) | All - First detection | 1.00 | - | - | - |
| Detection (*β*) | Kit & Juv - All farms - Each occasion | 1.00 | - | - | - |
| Detection (*β*) | Ad - Social - All farms | 1.00 | - | - | - |
| Detection (*β*) | Ad - Unsocial - Farm A & T | 0.93 | 0.85 | 0.97 | 0.03 |
| Detection (*β*) | Ad - Unsocial - Farm B | 0.81 | 0.67 | 0.90 | 0.06 |
| Detection (*β*) | Ad - Unsocial - Farm C & F | 0.72 | 0.58 | 0.82 | 0.06 |
| Detection (*β*) | Recovered - Farm A, C, F & T | 0.80 | 0.68 | 0.89 | 0.05 |
| Detection (*β*) | Recovered - Farm B | 0.12 | 0.03 | 0.37 | 0.08 |
| Age assignment (*α*) | Kit & Juv - Farm A & F | 0.84 | 0.73 | 0.91 | 0.04 |
| Age assignment (*α*) | Kit - Farm B | 0.91 | 0.58 | 0.99 | 0.08 |
| Age assignment (*α*) | Juv - Farm B | 0.56 | 0.33 | 0.77 | 0.12 |
| Age assignment (*α*) | All - Farm C & T + Ad - Farm A, B & F | 1.00 | 0.98 | 1.00 | 0.00 |
| Blood sample (*η*) | Kit -Unsociable - Spring - Farm A,B &T | 0.00 | 0.00 | 0.00 | 0.00 |
| Blood sample (*η*) | Juv - Unsociable - Spring & Autumn - Farm A, B &T | 0.94 | 0.80 | 0.99 | 0.04 |
| Blood sample (*η*) | Ad - Unsociable - Spring & Winter - Farm A, B & T | 0.74 | 0.67 | 0.81 | 0.04 |
| Blood sample (*η*) | Kit - Unsociable - Summer & Winter - Farm A,B &T | 0.44 | 0.24 | 0.66 | 0.11 |
| Blood sample (*η*) | Juv - Unsociable - Summer & Winter - Farm A, B &T | 0.76 | 0.56 | 0.89 | 0.08 |
| Blood sample (*η*) | Ad - Unsociable - Summer & Autumn - Farm A, B & T | 0.51 | 0.42 | 0.60 | 0.05 |
| Blood sample (*η*) | Kit - Unsociable - Autumn - Farm A,B&T | 0.11 | 0.04 | 0.29 | 0.06 |
| Blood sample (*η*) | Kit -Sociable - Spring - Farm A,B &T | 0.00 | 0.00 | 0.00 | 0.00 |
| Blood sample (*η*) | Juv - Sociable - Spring & Autumn - Farm A, B &T | 0.98 | 0.91 | 1.00 | 0.02 |
| Blood sample (*η*) | Ad - Sociable - Spring & Winter - Farm A, B & T | 0.89 | 0.83 | 0.93 | 0.03 |
| Blood sample (*η*) | Kit - Sociable - Summer & Winter - Farm A,B &T | 0.68 | 0.45 | 0.85 | 0.11 |
| Blood sample (*η*) | Juv - Sociable - Summer & Winter - Farm A, B &T | 0.90 | 0.76 | 0.96 | 0.05 |
| Blood sample (*η*) | Ad - Sociable - Summer & Autumn - Farm A, B & T | 0.74 | 0.65 | 0.82 | 0.04 |
| Blood sample (*η*) | Kit - Sociable - Autumn - Farm A,B&T | 0.25 | 0.09 | 0.53 | 0.12 |
| Blood sample (*η*) | Kit -Unsociable - Spring - Farm C | 0.00 | 0.00 | 0.00 | 0.00 |
| Blood sample (*η*) | Juv - Unsociable - Spring & Autumn - Farm C | 0.97 | 0.86 | 0.99 | 0.02 |
| Blood sample (*η*) | Ad - Unsociable - Spring & Winter - Farm C | 0.84 | 0.72 | 0.91 | 0.05 |
| Blood sample (*η*) | Kit - Unsociable - Summer & Winter - Farm C | 0.58 | 0.32 | 0.80 | 0.13 |
| Blood sample (*η*) | Juv - Unsociable - Summer & Winter - Farm C | 0.85 | 0.65 | 0.95 | 0.07 |
| Blood sample (*η*) | Ad - Unsociable - Summer & Autumn - Farm C | 0.65 | 0.48 | 0.79 | 0.08 |
| Blood sample (*η*) | Kit - Unsociable - Autumn - Farm C | 0.18 | 0.06 | 0.43 | 0.09 |
| Blood sample (*η*) | Kit -Sociable - Spring - Farm C | 0.00 | 0.00 | 0.00 | 0.00 |
| Blood sample (*η*) | Juv - Sociable - Spring & Autumn - Farm C | 0.99 | 0.94 | 1.00 | 0.01 |
| Blood sample (*η*) | Ad - Sociable - Spring & Winter - Farm C | 0.93 | 0.88 | 0.96 | 0.02 |
| Blood sample (*η*) | Kit - Sociable - Summer & Winter - Farm C | 0.79 | 0.57 | 0.91 | 0.09 |
| Blood sample (*η*) | Juv - Sociable - Summer & Winter - Farm C | 0.94 | 0.84 | 0.98 | 0.03 |
| Blood sample (*η*) | Ad - Sociable - Summer & Autumn - Farm C | 0.84 | 0.73 | 0.90 | 0.04 |
| Blood sample (*η*) | Kit - Sociable - Autumn - Farm C | 0.38 | 0.15 | 0.67 | 0.14 |
| Blood sample (*η*) | Kit -Unsociable - Spring - Farm F | 0.00 | 0.00 | 0.00 | 0.00 |
| Blood sample (*η*) | Juv - Unsociable - Spring & Autumn - Farm F | 0.88 | 0.62 | 0.97 | 0.08 |
| Blood sample (*η*) | Ad - Unsociable - Spring & Winter - Farm F | 0.55 | 0.43 | 0.67 | 0.06 |
| Blood sample (*η*) | Kit - Unsociable - Summer & Winter - Farm F | 0.25 | 0.11 | 0.46 | 0.09 |
| Blood sample (*η*) | Juv - Unsociable - Summer & Winter - Farm F | 0.57 | 0.33 | 0.79 | 0.13 |
| Blood sample (*η*) | Ad - Unsociable - Summer & Autumn - Farm F | 0.31 | 0.21 | 0.43 | 0.06 |
| Blood sample (*η*) | Kit - Unsociable - Autumn - Farm F | 0.05 | 0.01 | 0.16 | 0.03 |
| Blood sample (*η*) | Kit -Sociable - Spring - Farm F | 0.00 | 0.00 | 0.00 | 0.00 |
| Blood sample (*η*) | Juv - Sociable - Spring & Autumn - Farm F | 0.95 | 0.81 | 0.99 | 0.04 |
| Blood sample (*η*) | Ad - Sociable - Spring & Winter - Farm F | 0.77 | 0.67 | 0.84 | 0.04 |
| Blood sample (*η*) | Kit - Sociable - Summer & Winter - Farm F | 0.47 | 0.26 | 0.70 | 0.12 |
| Blood sample (*η*) | Juv - Sociable - Summer & Winter - Farm F | 0.78 | 0.57 | 0.91 | 0.09 |
| Blood sample (*η*) | Ad - Sociable - Summer & Autumn - Farm F | 0.55 | 0.43 | 0.66 | 0.06 |
| Blood sample (*η*) | Kit - Sociable - Autumn - Farm F | 0.12 | 0.04 | 0.33 | 0.07 |
| Serological results (*τ*) | Negative - Titre = 0 - Occasions 1-3 | 0.93 | 0.82 | 0.98 | 0.04 |
| Serological results (*τ*) | Negative - 0 < Titre < 25 - Occasions 1-3 | 0.04 | 0.01 | 0.11 | 0.02 |
| Serological results (*τ*) | Negative - Titre ≥ 25 - Occasions 1-3 | 0.03 | NA | NA | NA |
| Serological results (*τ*) | Negative - Titre = 0 - Occasions 4-8 | 0.96 | 0.88 | 0.99 | 0.02 |
| Serological results (*τ*) | Negative - 0 < Titre < 25 - Occasions 4-8 | 0.04 | 0.01 | 0.12 | 0.02 |
| Serological results (*τ*) | Negative - Titre ≥ 25 - Occasions 4-8 | 0.00 | 0.00 | 0.00 | 0.00 |
| Serological results (*τ*) | Positive - Titre = 0 - Farm A, B & F - Occasions 1-3 | 0.24 | NA | NA | NA |
| Serological results (*τ*) | Positive - 0<Titre<25 - Farm A, B & F - Occasions 1-3 | 0.05 | 0.02 | 0.09 | 0.02 |
| Serological results (*τ*) | Positive - Titre ≥ 25 - Farm A, B & F - Occasions 1-3 | 0.71 | 0.43 | 0.89 | 0.12 |
| Serological results (*τ*) | Positive - Titre = 0 - Farm A, B & F - Occasions 4-8 | 0.00 | 0.00 | 0.00 | 0.00 |
| Serological results (*τ*) | Positive - 0<Titre<25 - Farm A, B & F - Occasions 4-8 | 0.06 | 0.03 | 0.12 | 0.02 |
| Serological results (*τ*) | Positive - Titre ≥ 25 - Farm A, B & F - Occasions 4-8 | 0.94 | 0.88 | 0.97 | 0.02 |
| Serological results (*τ*) | Positive - Titre = 0 - Farm C & T - Occasions 1-3 | 0.30 | NA | NA | NA |
| Serological results (*τ*) | Positive - 0<Titre<25 - Farm C & T - Occasions 1-3 | 0.23 | 0.14 | 0.35 | 0.06 |
| Serological results (*τ*) | Positive - Titre ≥ 25 - Farm C & T - Occasions 1-3 | 0.47 | 0.31 | 0.64 | 0.09 |
| Serological results (*τ*) | Positive - Titre = 0 - Farm C & T - Occasions 4-8 | 0.00 | 0.00 | 0.00 | 0.00 |
| Serological results (*τ*) | Positive - 0<Titre<25 - Farm C & T - Occasions 4-8 | 0.32 | 0.22 | 0.45 | 0.06 |
| Serological results (*τ*) | Positive - Titre ≥ 25 - Farm C & T - Occasions 4-8 | 0.68 | 0.55 | 0.78 | 0.06 |

**References**

Choquet, R., Rouan, L., Pradel, R., 2009. Program E-SURGE, a software application for fitting multi-event models. Environmental and Ecological Statistics, 3, 845–865.

Choquet, R., 2007. E-SURGE 1-0 user’s manual. CEFE, UMR 5175, Montpellier, France.(<http://ftp.cefe.cnrs.fr/biom/soft-cr/>).
