# Supplementary material for: A multi-event capture-recapture analysis of Toxoplasma gondii seroconversion dynamics in farm cats
Source: Parasit Vectors. 2018 Jun 8;11:339. doi: 10.1186/s13071-018-2834-4 (PMC5994099; doi:10.1186/s13071-018-2834-4)
Supplement: Supplementary file 2 — Table S3. Dataset showing Toxoplasma gondii antibody titres obtained from the modified agglutinated test (MAT) and the characteristics of the sampled cat. (DOCX 51 kb) [file 13071_2018_2834_MOESM2_ESM.docx]

**Additional file 2: Details of the dataset**

This file presents the details of the dataset showing *Toxoplasma gondii* antibody titres obtained from the modified agglutinated test (MAT) and the characteristics of the sampled cat.

**Table S3**: Dataset showing *Toxoplasma gondii* antibody titres obtained from the modified agglutinated test (MAT) and the characteristics of the sampled cat. Dash indicates sampling session when a cat was detected but blood was not collected and thus not tested for the presence of *T. gondii* antibodies. Abbreviations: D, found dead after the previous session; A, farm A; B, farm B; C, farm C; F, farm F; T, farm T; M, male; F, female; S, sociable cat; U, unsociable cat.

|  | **Sampling sessions** | | | | | | | | **Groups** | | |
| --- | --- | --- | --- | --- | --- | --- | --- | --- | --- | --- | --- |
| **Cat** | **1** | **2** | **3** | **4** | **5** | **6** | **7** | **8** | **Farm** | **Gender** | **Sociability** |
| **AC01** | 1600 |  |  | 1600 | - | 800 | - | D | A | M | U |
| **AC02** | 50 |  | - |  | - | - | - | 100 | A | F | U |
| **AC03** | 50 | - | - | 400 | - |  | 400 | 800 | A | M | U |
| **AC04** | 6 | - | - | 100 | 200 | - | - | 100 | A | M | U |
| **AC05** | 0 | 0 | 0 | 10 | 25 | - | D |  | A | F | U |
| **AC06** | 400 | - | - | 400 | 200 | 1600 | 1600 | 1600 | A | F | S |
| **AC07** | 0 | 0 | 100 | 25000 | 12800 |  |  |  | A | M | S |
| **AC08** | 25 | 0 | 400 | 200 | 100 | D |  |  | A | F | S |
| **AC09** | 10 | - | 0 | - | 6 | - | - | - | A | F | U |
| **AC10** | 25 | D |  |  |  |  |  |  | A | M | U |
| **AC11** | 200 | 6400 | 3200 | 3200 | - | 3200 | - | - | A | F | S |
| **AC12** | - | 0 | 0 | 50 | 25 | 10 | 50 | 25 | A | F | S |
| **AC13** |  | 0 | D |  |  |  |  |  | A | M | S |
| **AC14** | - | 0 | 0 | - |  |  |  |  | A | F | U |
| **AC15** |  |  | 0 | 200 | 400 | 6400 | 12800 | 6400 | A | F | S |
| **AC16** |  |  | 3200 | D |  |  |  |  | A | F | U |
| **AC17** |  |  | 0 | 100 | 50 |  |  |  | A | F | S |
| **AC18** |  |  | 3200 | 3200 | D |  |  |  | A | M | U |
| **AC19** |  |  | 0 | 25 | 10 | 10 | D |  | A | F | S |
| **AC20** |  |  | 800 | 400 | 400 | D |  |  | A | M | U |
| **AC21** |  |  | 6400 | D |  |  |  |  | A | F | U |
| **AC22** |  |  |  | 50 | 1600 | 800 | 100 | 400 | A | M | U |
| **AC23** |  |  | - | 0 | 0 | 12800 | 12800 | 12800 | A | M | S |
| **AC24** |  |  | - | 800 | 200 | 10 | 200 | 200 | A | M | S |
| **AC25** |  |  |  | 0 | D |  |  |  | A | M | U |
| **AC26** | - | - | - | 100 |  | - | - | - | A | M | U |
| **AC27** |  |  |  | 100 | - | 400 |  | - | A | M | U |
| **AC28** |  |  |  | 0 | 0 | 1600 | 1600 | 400 | A | F | S |
| **AC29** |  |  |  |  |  | 0 | D |  | A | F | U |
| **AC30** |  |  |  |  |  | 1600 | 3200 | - | A | F | U |
| **AC31** |  |  |  |  |  | 400 | 3200 | D | A | M | U |
| **AC32** |  |  |  |  |  | 0 | 25 | 12800 | A | M | S |
| **AC33** |  |  |  |  | - | 0 | 0 | 0 | A | M | S |
| **AC34** |  |  |  |  |  | 25 |  |  | A | M | S |
| **AC35** |  |  |  |  |  |  | 0 | 3200 | A | M | S |
| **AC36** |  |  |  |  |  |  | 800 | 400 | A | M | U |
| **AC37** |  |  |  |  |  | - | 100 | 400 | A | F | U |
| **AC38** |  |  |  |  |  |  | 0 | D | A | M | S |
| **AC39** |  |  |  |  |  | - | 50 | 50 | A | M | U |
| **AC40** |  |  |  |  |  | - | 0 | D | A | F | U |
| **AC41** |  |  |  |  |  |  | 0 | 25 | A | F | S |
| **AC42** |  | - | D |  |  |  |  |  | A | F | S |
| **AC43** |  |  | - | D |  |  |  |  | A | F | U |
| **AC44** |  |  | - | D |  |  |  |  | A | F | U |
| **BC01** |  |  | 25 | 10 | 25 | - | - | 100 | B | M | U |
| **BC02** |  |  | 0 | 25 |  |  | - | 50 | B | F | U |
| **BC03** |  |  | 200 |  |  |  | 800 | - | B | F | U |
| **BC04** |  |  | 50 | 50 |  |  |  |  | B | M | U |
| **BC05** |  |  | 400 | 100 | 100 | D |  |  | B | F | S |
| **BC06** |  |  | 0 | 0 | - |  |  |  | B | F | U |
| **BC07** |  |  | 0 | 0 | - |  |  |  | B | F | U |
| **BC08** |  |  | 0 | D |  |  |  |  | B | F | U |
| **BC09** |  |  | 0 | 0 | - |  | 0 | 0 | B | F | U |
| **BC10** |  |  | 25 |  | 25 |  | - |  | B | F | U |
| **BC11** |  |  | 0 | 0 | - |  | - | 200 | B | F | U |
| **BC12** |  |  | 0 | 0 |  |  |  |  | B | F | U |
| **BC13** |  |  |  | 0 | 0 | - | 0 | 0 | B | F | U |
| **BC14** |  |  |  | 0 | 0 |  |  |  | B | F | U |
| **BC15** |  |  |  | 100 |  |  |  |  | B | M | U |
| **BC16** |  |  | - | 0 | 0 | - | - | - | B | M | U |
| **BC17** |  |  | - | 0 | 0 | - | 0 | - | B | F | U |
| **BC18** |  |  | - | 0 | 0 |  |  |  | B | M | U |
| **BC19** |  |  | - | 0 | - | 0 | 0 | - | B | F | U |
| **BC20** |  |  |  | 400 |  |  |  |  | B | M | U |
| **BC21** |  |  | - | 0 | - |  |  |  | B | F | U |
| **BC22** |  |  | - | 0 | - |  |  |  | B | M | U |
| **BC23** |  |  | - | 3200 | 6400 | 6400 | - | 6400 | B | F | U |
| **BC24** |  |  |  |  |  |  | 0 | - | B | M | U |
| **BC25** |  |  |  |  |  | - | 0 | 0 | B | F | U |
| **BC26** |  |  |  |  |  |  |  | 0 | B | F | U |
| **BC27** |  |  |  |  |  |  |  | 0 | B | F | U |
| **BC28** |  |  | - | - |  |  |  |  | B | M | U |
| **BC29** |  |  | - |  |  |  |  |  | B | M | U |
| **BC30** |  |  | - |  |  |  |  |  | B | M | U |
| **BC31** |  |  | - |  |  |  |  |  | B | F | U |
| **BC32** |  |  | - |  |  |  |  |  | B | F | U |
| **CC01** | 50 | - | 200 | 10 | 400 | 200 | 100 | 200 | C | M | S |
| **CC02** | 0 | 0 | 0 | 0 | 0 | - | 0 | 0 | C | F | S |
| **CC03** | 0 | 0 | 0 | 10 | 10 | 50 | D |  | C | M | S |
| **CC04** | 10 | 10 | 0 | 25 | 10 | 50 | 50 | - | C | M | S |
| **CC05** | 0 |  | 10 |  |  |  |  |  | C | M | U |
| **CC06** | 10 | 10 | - | 25 | - | D |  |  | C | M | S |
| **CC07** | 10 | 6 | D |  |  |  |  |  | C | F | U |
| **CC08** | 100 | - | - | - | - | D |  |  | C | M | S |
| **CC09** | 50 | - | - | 10 | D |  |  |  | C | F | S |
| **CC10** | 0 |  |  |  |  |  |  |  | C | M | U |
| **CC11** | 50 | - | - | 25 | 50 | D |  |  | C | F | S |
| **CC12** | 50 |  |  |  |  |  |  |  | C | F | S |
| **CC13** | - | D |  |  |  |  |  |  | C | M | U |
| **CC14** | - | 0 | 0 | 0 |  | 0 | 0 | 0 | C | F | U |
| **CC15** | - | 0 | 0 | 0 | 0 |  |  |  | C | F | U |
| **CC16** | - | 0 | 0 | 0 | 0 | 0 | 0 | 0 | C | M | S |
| **CC17** |  | 0 | 50 | 25 | 50 | 50 | 50 | 100 | C | M | S |
| **CC18** |  | 0 | 0 | 10 | 10 | 100 | 50 | 25 | C | M | S |
| **CC19** |  | 0 | 0 | 10 | 50 | 100 | 200 | 200 | C | M | S |
| **CC20** |  |  | 0 | D |  |  |  |  | C | F | U |
| **CC21** |  |  | 0 | D |  |  |  |  | C | F | S |
| **CC22** | - |  |  |  |  |  |  |  | C | F | S |
| **CC23** |  |  | - | D |  |  |  |  | C | M | S |
| **CC24** |  |  | - | D |  |  |  |  | C | M | S |
| **CC25** |  |  | - | D |  |  |  |  | C | F | S |
| **CC26** |  |  |  |  |  | 0 | 0 | 0 | C | F | S |
| **CC27** |  |  |  |  |  | 0 | 0 | 0 | C | F | S |
| **CC28** |  |  |  |  |  |  | 0 | D | C | F | S |
| **CC29** |  |  |  |  |  | - | D |  | C | F | U |
| **FC01** | 3200 | - | - | 6400 | 6400 | 1600 | 6400 | D | F | F | S |
| **FC02** | 800 | - | - | 1600 | 800 | 400 | 800 | 400 | F | M | S |
| **FC03** | 10 | - | - | 25 | 50 | 3200 | 50 | 100 | F | F | S |
| **FC04** | 0 | 0 | 0 | - | - | - | - | - | F | M | U |
| **FC05** | 6400 | - | D |  |  |  |  |  | F | F | S |
| **FC06** | 400 | - | - | - | - | - | - | D | F | F | S |
| **FC07** | 0 | - | 25000 | 6400 | - | - | - | 12800 | F | F | U |
| **FC08** | 0 | - |  |  | - | - |  |  | F | F | U |
| **FC09** | 400 |  | - | - | D |  |  |  | F | M | U |
| **FC10** | 100 | - | - | 400 | 400 | 1600 | 1600 | - | F | F | S |
| **FC11** | 0 |  |  | 1600 | 6400 | - |  |  | F | M | U |
| **FC12** | 100 | - | - | 6400 | 800 | 6400 | 1600 | 800 | F | M | S |
| **FC13** | - | 0 | 0 | 400 | 400 | 400 | 200 | 100 | F | M | S |
| **FC14** | - | 0 | 25 | 10 | 25 | 25 | 100 | 100 | F | F | S |
| **FC15** | - | D |  |  |  |  |  |  | F | M | S |
| **FC16** | - | D |  |  |  |  |  |  | F | M | S |
| **FC17** | - | D |  |  |  |  |  |  | F | F | S |
| **FC18** | - | D |  |  |  |  |  |  | F | F | S |
| **FC21** | - | 800 | - | - | - | - |  |  | F | M | U |
| **FC22** | - |  |  | 0 | - |  | 3200 |  | F | M | U |
| **FC23** |  |  |  |  | 200 | - | - | - | F | F | U |
| **FC25** |  |  |  | - | 400 | 3200 | 1600 | 400 | F | M | S |
| **FC26** |  |  |  | - | 50 | 3200 | - | - | F | F | U |
| **FC27** |  |  |  |  |  | 0 | 0 | 0 | F | F | U |
| **FC28** |  |  |  |  |  | - | 0 | D | F | M | S |
| **FC29** |  |  |  |  |  | - | 200 | 12800 | F | M | S |
| **FC30** |  |  |  |  |  | - | 0 | D | F | M | U |
| **FC31** |  |  |  |  |  | - | 100 | 100 | F | F | U |
| **FC32** |  |  |  |  |  |  | 0 |  | F | F | U |
| **FC33** |  |  |  |  |  |  | 50 | - | F | F | U |
| **FC34** |  |  |  |  |  | - | - |  | F | M | U |
| **FC35** |  |  |  |  |  | - | D |  | F | M | U |
| **FC36** |  |  |  |  |  | - | D |  | F | F | U |
| **TC01** | 0 | 0 | 0 | 0 | 0 | D |  |  | T | M | S |
| **TC02** | 10 | - |  |  |  |  |  |  | T | M | U |
| **TC03** | 0 | 0 | - | - | - | - | - | - | T | M | S |
| **TC04** | 0 | D |  |  |  |  |  |  | T | F | S |
| **TC05** | 0 | 0 | - | 0 | 0 | - | 0 | 25 | T | M | U |
| **TC06** | 0 | 0 | 50 | 0 | 25 | 25 | 25 | 25 | T | F | S |
| **TC07** | 0 | - | 0 | 0 | 10 | 10 | 0 | 0 | T | M | U |
| **TC08** | 0 |  | - | 10 | - | 25 | 100 | 25 | T | F | U |
| **TC09** | 0 | 0 | 0 | 0 | 50 | 10 | 10 | 10 | T | M | U |
| **TC10** | 0 | - | - | - | D |  |  |  | T | F | U |
| **TC13** | - | D |  |  |  |  |  |  | T | F | S |
| **TC14** | - | D |  |  |  |  |  |  | T | M | S |
| **TC15** | - | - | - | - |  |  |  |  | T | F | S |
| **TC16** | - | 0 | 0 | 0 | 0 | 0 | 0 | 0 | T | M | S |
| **TC17** | - | - | 0 | D |  |  |  |  | T | M | S |
| **TC18** |  |  | 0 | - | 10 | D |  |  | T | F | S |

The data recorded in the field and presented in Table S3 was converted into 15 events to perform multi-event capture–recapture analysis: 0, undetected; 1, kitten tested with titre = 0; 2, kitten tested with titre ≥ 25; 3, kitten detected but not tested; 4, juvenile tested with titre = 0; 5, juvenile tested with titre ≥25; 6, juvenile detected but not tested; 7, adult tested with titre = 0; 8, adult tested with 0 < titre < 25; 9, adult tested with titre ≥ 25; 10, adult detected but not tested; 11, individual of undetermined age tested with titre = 0; 12, individual of undetermined age tested with titre ≥ 25; 13, individual of undetermined age detected but not tested; and 14, cat recovered dead. Twenty groups of cats were defined in combinations of the farm of origin (A, B, C, F and T), gender (male ‘M’ or female ‘F’) and sociability (sociable ‘S’ or unsociable ‘U’).
