# Supplementary material for: A multi-event capture-recapture analysis of Toxoplasma gondii seroconversion dynamics in farm cats
Source: Parasit Vectors. 2018 Jun 8;11:339. doi: 10.1186/s13071-018-2834-4 (PMC5994099; doi:10.1186/s13071-018-2834-4)
Supplement: Supplementary file 3 — Description of survival rates, detection rates, age assignment and blood collection rates estimated from the best retained model. (DOCX 12 kb) [file 13071_2018_2834_MOESM3_ESM.docx]

**Additional file 3: Estimated survival, detection, age assignment and blood collection rates.**

This file provides a description of survival rates, detection rates, age assignment and blood collection rates estimated from the best retained model.

In the best retained model, the cat survival rate (*S*) was variable among age classes and seasons (Additional file 1: Table S2). For all three age classes, survival rates were lower between January and April than in other seasons. Survival rates increased with age: *S*=0.44 [0.29–0.61] and *S*=0.63 [0.51–0.74] for kittens, *S*=0.71 [0.50–0.86] and *S*=0.84 [0.72–0.92] for juveniles, *S*=0.84 [0.77–0.89] and *S*=0.92 [0.88–0.95] for adults in spring and other seasons respectively.

Kittens and juveniles were systematically detected at all sessions (detection rate *β*=1). However, detection rates of adults varied according to sociability towards humans and depending on the farm (Additional file 1: Table S2). Detection of unsociable cats varied from *β*=0.72 [0.58–0.82] to *β*=0.93 [0.85–0.97] depending on the farm, whereas sociable cats were systematically detected (*β*=1). The age assignment (α) of detected cats was conditional on the age-class and the farm. When detected, adults were systematically correctly assigned to their true age-class (*α*=1 [0.98–1.00]) regardless of the farm. The rate of correct assignment for kittens and juveniles varied between *α*=0.56 [0.33–077] and *α*=1 [.98–1] depending on the farm (Additional file 1: Table S2).

Blood collection rates were highly variable (*η*=0 to *η*=0.99 [0.94–1.00]) according to age-class, season, population and sociability. All the estimated parameters for blood collection are presented in Table S2 in Additional file 1. To summarise, estimated blood collection rates varied among the five studied farms, but a similar pattern was observed: collection rates were higher for kittens in summer and winter (from *η*=0.25 [0.11–0.46] to *η*=0.79 [0.57–0.91]) compared to autumn (from *η*=0.05 [0.01–0.16] to *η*=0.38 [0.15–0.66]), whereas no blood samples were taken from kittens in spring. Higher rates of blood samples were collected in juveniles in spring and autumn (from *η*=0.88 [0.62–0.97] to *η*=0.99 [0.94–1.00]) compared to summer and winter (from *η*=0.57 [0.33–0.79] to *η*=0.94 [0.84–0.98]). Blood sample rates also varied in adults and were higher in spring and winter (from *η*=0.55 [0.43–0.67] to *η*=0.93 [0.88–0.96]) than in summer and autumn (from *η*=0.31 [0.21–0.43] to *η*=0.83 [0.73–0.90]). Sociability also influenced blood collection rates: blood was collected from cats caught by hand more often than from trapped cats.
